# An Oct4-Centered Protein Interaction Network in Embryonic Stem Cells

**DOI:** 10.1016/j.stem.2010.02.014

**Published:** 2010-04-02

**Authors:** Debbie L.C. van den Berg, Tim Snoek, Nick P. Mullin, Adam Yates, Karel Bezstarosti, Jeroen Demmers, Ian Chambers, Raymond A. Poot

**Affiliations:** 1 Department of Cell Biology, Erasmus MC, Dr. Molewaterplein 50, 3015GE Rotterdam, The Netherlands; 2 Proteomics Center, Erasmus MC, Dr. Molewaterplein 50, 3015GE Rotterdam, The Netherlands; 3 MRC Centre for Regenerative Medicine, Institute for Stem Cell Research, School of Biological Sciences, University of Edinburgh, King's Buildings, West Mains Road, Edinburgh EH9 3JQ, UK

**Keywords:** STEMCELL

## Abstract

Transcription factors, such as Oct4, are critical for establishing and maintaining pluripotent cell identity. Whereas the genomic locations of several pluripotency transcription factors have been reported, the spectrum of their interaction partners is underexplored. Here, we use an improved affinity protocol to purify Oct4-interacting proteins from mouse embryonic stem cells (ESCs). Subsequent purification of Oct4 partners Sall4, Tcfcp2l1, Dax1, and Esrrb resulted in an Oct4 interactome of 166 proteins, including transcription factors and chromatin-modifying complexes with documented roles in self-renewal, but also many factors not previously associated with the ESC network. We find that Esrrb associated with the basal transcription machinery and also detect interactions between transcription factors and components of the TGF-β, Notch, and Wnt signaling pathways. Acute depletion of Oct4 reduced binding of Tcfcp2l1, Dax1, and Esrrb to several target genes. In conclusion, our purification protocol allowed us to bring greater definition to the circuitry controlling pluripotent cell identity.

## Highlights

► An improved affinity method identified an Oct4 interactome of >160 proteins ► Iterative purification with Oct4 interactors helped define the network ► Network shows links to the basal transcription machinery and signaling pathways ► Transcription factor interactions may facilitate recruitment to the genome

## Introduction

Embryonic stem cells (ESCs) are derived from the inner cell mass of mammalian embryos and have the unique ability to grow indefinitely in culture while retaining their pluripotency (Smith, 2001). This self-renewal capacity is regulated by a set of transcription factors including Oct4, Nanog, and Sox2 (Niwa, 2007). ESCs are particularly sensitive to dosage alterations in Oct4; a 50% increase or decrease in the level of Oct4 causes differentiation into cells expressing markers of endoderm and mesoderm or trophectoderm, respectively (Niwa et al., 2000). Oct4 also plays a central role in the reprogramming of both human and mouse fibroblasts into induced pluripotent stem cells (iPSCs) (Okita et al., 2007; Takahashi and Yamanaka, 2006; Wernig et al., 2007). Oct4 is one of a set of reprogramming factors that usually also includes Sox2, Klf4, and c-*myc* (Hochedlinger and Plath, 2009; Yamanaka, 2009). Sox2, Klf4, and c-*myc* can be replaced by family members such as Sox1, Sox3, Klf2, Klf5, L-Myc, and N-Myc, but without Oct4 no reprogramming occurs (Nakagawa et al., 2008).

Recently, genome-wide chromatin immunoprecipitation (ChIP) analyses in mouse ESCs have identified the genomic binding sites of Oct4 and a number of other ESC transcription factors (Chen et al., 2008b; Kim et al., 2008; Sridharan et al., 2009). Oct4 clusters with a variable but overlapping set of transcription factors at many genomic locations, including promoters and enhancers (reviewed in Chambers and Tomlinson, 2009). Clusters with a relatively high number of different transcription factors appear to correlate with ESC-specific expression of the nearby gene (Chen et al., 2008b; Kim et al., 2008). The mechanism for this molecular clustering may have similarities with the partnership of Oct4 with Sox2. Oct4 and Sox2 have low affinity for each other in solution (Ambrosetti et al., 1997; Wissmüller et al., 2006), yet this affinity is critical for the cooperative binding of Oct and Sox proteins to adjacent sites on DNA (Ambrosetti et al., 1997; Reményi et al., 2003). Therefore, identifying the interaction partners of transcription factors important for pluripotency could add novel components to the pluripotency transcriptional network and help to elucidate the assembly mechanism of transcription factor clusters. However, physical interactions between ESC transcription factors remain underinvestigated. Low-affinity interactions between transcription factors together with the generation of sufficient ESC material for biochemical purification complicate an effective search for interaction partners. To address these drawbacks, we improved the FLAG-affinity-based protein purification protocol. By using only small amounts of starting material, we initially purified FLAG-tagged Oct4 and its interacting proteins from mouse ESCs. Subsequently, we purified four of the identified Oct4-interacting ESC transcription factors: Sall4, Esrrb, Dax1, and Tcfcp2l1. The resulting interaction network contains many transcriptional regulators and chromatin-modifying complexes known to play roles in ESC self-renewal, as well as transcriptional regulators not previously affiliated with pluripotency. We find associations between transcription factors and several signaling pathways and identify a physical connection between the ESC transcription factor Esrrb and the basal transcription machinery. Thus, our methodology allowed for a much more detailed view of the physical interactions between factors that act in the ESC pluripotency network.

## Results

### Purification of Oct4-Interacting Proteins from ESCs

**Figure 1:**
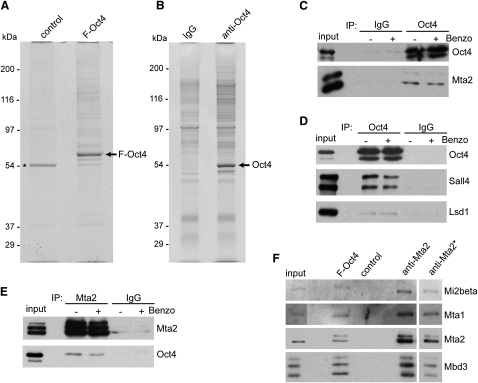
Purification of Oct4 and Its Interacting Proteins (A) Colloidal Coomassie-stained SDS-polyacrylamide gel of a FLAG-Oct4 (F-Oct4) and control purification. Asterisk indicates contaminating band. The F-Oct4 band is indicated. (B) Colloidal Coomassie-stained SDS-polyacrylamide gel of immunoprecipitated endogenous Oct4 and a control immunoprecipitation via IgG. The Oct4 band is indicated. (C and D) Oct4 immunoprecipitates analyzed by western blots with the indicated antibodies. Benzonase (Benzo) was added where indicated. (E) MTA2 immunoprecipitates analyzed by western blots with the indicated antibodies. (F) Subunit stoichiometry of F-Oct4-bound NuRD complex (F-Oct4) compared to anti-Mta2 coimmunoprecipitated NuRD complex (anti-Mta2) by western blot against the indicated NuRD subunits. Asterisk indicates a lighter exposure of the same experiment. See also Figure S1.

We have previously described a mouse ESC line in which, under self-renewing conditions, all the Oct4 protein in the cell has an N-terminal triple FLAG-tag (F-Oct4) (van den Berg et al., 2008). Both F-Oct4 and the parental ZHBTc4 cells have a normal ESC morphology (Niwa et al., 2000; van den Berg et al., 2008) and express normal levels of ESC markers Sox2, Sall4 (Figure S1A available online), Klf4, Dax1, Zfp42, and Eras (Figure S1B). This indicates that the F-Oct4 protein present in the F-Oct4 cells maintains their ESC identity. We prepared nuclear extracts from F-Oct4 cells and ZHBTc4 cells, which do not express F-Oct4 and serve as a control. FLAG-affinity purifications were performed from 1.5 ml of nuclear extract (equivalent to ∼4 × 10^8^ cells) with an improved protocol in which near-physiological salt conditions, low detergent concentrations, and low-adherence tubes were employed (see Experimental Procedures for details). Benzonase nuclease was added to the extract to remove the remaining DNA (Figure S1C), thereby eliminating protein interactions mediated indirectly by DNA bridging. Virtually all F-Oct4 in the extract was bound to the FLAG-antibody beads and subsequently eluted by FLAG peptide competition (Figure S1D). An SDS polyacrylamide gel of the eluted fractions, stained with a sensitive Colloidal Coomassie protocol, showed Oct4 as the predominant band in the F-Oct4 sample (Figure 1A). The control sample showed only one prominent band, which was also present in the F-Oct4 sample but was otherwise devoid of major contaminants. This indicates that our FLAG-mediated purification of Oct4 has a very good signal to background ratio. The presence of multiple bands of lower intensity in the F-Oct4 lane suggests that Oct4 interacts with a variety of proteins at substoichiometric levels. The majority of Oct4 runs at approximately its own molecular weight on a gel filtration column (Figure S1E), unlike a stable complex such as NuRD. Therefore, most Oct4 interactions are likely to be weak and do not survive the 4 hr gel filtration procedure, in which dissociation causes an irreversible loss of the interaction. To independently verify candidate F-Oct4-interacting proteins, we also immunoprecipitated endogenous Oct4 from nuclear extracts of a different ESC line, 46C (Ying et al., 2003), with an antibody that captured all Oct4 from the extract (Figure S1F). Although we used the same buffer conditions and low-adherence tubes, this procedure gives higher background compared to the FLAG-affinity purification (Figure 1B), because proteins that bind nonspecifically to the antibody beads or the tubes cannot be excluded from the eluate by FLAG-peptide elution, as they can in the FLAG purification strategy.

We analyzed three independent F-Oct4 purifications and the endogenous Oct4 immunoprecipitation by mass spectrometry (Table 1). A representation of the identified proteins by a more quantitative measure, emPAI score (Ishihama et al., 2005), is shown in Table S1. Our list of more than 50 putative Oct4-associated proteins (Table 1) contains 22 transcription factors of which half have a role in maintaining pluripotency (Table 2). These include Sall4, Klf5, Zfp143, Esrrb, and Sox2, the best-characterized Oct4 partner for which 3D structures of the Oct4-Sox2-DNA ternary complex have been reported (Reményi et al., 2003; Williams et al., 2004). We also identified a number of chromatin-modifying complexes (CMCs). All of the subunits of the transcriptional repressor NuRD were specifically present, except for Rbbp4 (high background prevented inclusion of Rbbp4 in Table 1). We detected subunits from the chromatin-remodeling complexes SWI/SNF and Trrap/p400, the Lsd1 histone demethylase complex, and components of the polycomb repression complex 1 (PRC1).

Next we examined the presence of some of the identified interactors in Oct4 immunoprecipitates by immunoblotting. Indeed, we find that NuRD subunit Mta2 (Figure 1C), spalt-like protein Sall4, histone-demethylase Lsd1 (Figure 1D), Sall1, and Wdr5 (Figure S1G) coprecipitate with Oct4, whereas immunoprecipitates of Mta2 (Figure 1E) and Wdr5 (Figure S1H) contain Oct4. Recently, it was suggested that a subset of the NuRD subunits (Mta1 and 2, Gatad2a and Gatad2b, Hdac1 and 2) forms an Oct4/Nanog-associated complex called NODE (Nanog- and Oct4-associated deacetylase; Liang et al., 2008). We found that Oct4 binds the classical NuRD complex, as it was originally defined (Zhang et al., 1999), including catalytic subunit Mi2β and Mbd3 and Rbbp7 (Table 1). Immunoblotting confirmed this; the proportionate amount of antigen detected for Mi2β, Mbd3, Mta1, and Mta2 was the same in FLAG-Oct4 and Mta2 IP samples (Figure 1F). This suggests that Oct4-bound NuRD is similar or identical to classical NuRD in its composition and argues against the existence of Oct4-bound NuRD subcomplexes, such as NODE.

### Oct4-Interacting Proteins Correlate with Gene Regulation by Oct4 and ESC Self-Renewal

Proteins that interact with Oct4 may be expected to be Oct4 cofactors in gene regulation and have DNA binding profiles that overlap with Oct4. Recently, two studies reported the genome-wide binding sites of different sets of ESC transcription factors (Chen et al., 2008b; Kim et al., 2008). Five of the Oct4-interacting transcription factors identified here (Sox2, Nac1, Tcfcp2l1, Esrrb, Dax1) were investigated in those studies and were found to colocalize frequently with Oct4 (Table 2), including at the promoters of important pluripotency genes such as *Nanog* and *Oct4* (Chen et al., 2008b; Kim et al., 2008; Levasseur et al., 2008).

Phenotypes are documented for ∼60% of the identified Oct4-interacting proteins (Table 2). Of these, ∼65% (21/32) of the tested factors (Table 2) affect the ability of ESCs to remain undifferentiated. This includes most of the aforementioned transcription factors and subunits of all the identified Oct4-associated chromatin-modifying complexes (Table 2), except for the Lsd1 complex.

We then investigated whether genes encoding Oct4-interacting proteins are bound and regulated by Oct4. Gene expression profiling data from ZHBTc4 ESCs, which express Oct4 from a doxycycline-repressible transgene (Sharov et al., 2008), was combined with two different sets of Oct4 ChIP data (Chen et al., 2008b; Kim et al., 2008). We find that 14 factors (26%) are encoded by genes bound by Oct4 that are downregulated after 48 hr of doxycycline treatment (Table 2). This correlation of Oct4 binding and transcriptional regulation by Oct4 increases the interdependence of the associated proteins with Oct4, as previously observed (Wang et al., 2006).

### Purification of Interaction Partners of Sall4, Esrrb, Dax1, and Tcfcp2l1

**Figure 2:**
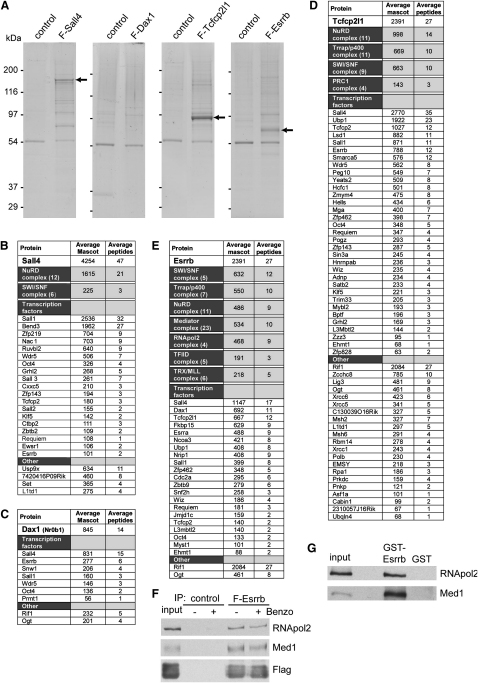
Purification of F-Sall4, F-Dax1, F-Tcfcp2l1, F-Esrrb, and Their Interacting Proteins (A) Colloidal Coomassie-stained SDS-polyacrylamide gels of representative purifications of the FLAG-tagged transcription factors and control purifications from the parental ESC line. Arrows indicate the respective FLAG-tagged proteins. (B–E) Summaries of the identified interacting proteins. The average Mascot score and number of identified unique peptides of two purifications without doxycycline addition are indicated for individual proteins or complexes. The number of identified subunits of a complex is between brackets. (F) F-Esrrb or control purifications analyzed by western blots with the indicated antibodies. Benzonase was added where indicated. (G) GST-Esrrb pull-downs analyzed by western blots with the indicated antibodies. Figure S3H (right) shows the purified GST proteins on a Coomassie-stained polyacrylamide gel. See also Figure S2 and Tables S2–S9.

Having established that our FLAG-affinity purification protocol identifies novel interactions that are independently verifiable and biologically relevant, an expanded network of Oct4 interactions was sought. Sall4, Esrrb, Dax1, and Tcfcp2l1 were selected for purification because of their consistent presence in all Oct4 purifications (Table 1). The spalt-like transcription factor Sall4 is important for stabilizing ESC self-renewal (Yuri et al., 2009; Zhang et al., 2006). Orphan receptor Esrrb is important for ESC self-renewal (Ivanova et al., 2006; Loh et al., 2006). Esrrb positively regulates the expression of key pluripotency gene *Nanog* (van den Berg et al., 2008), and overexpression of Esrrb allows short-term ESC maintenance without the addition of exogenous LIF (Zhang et al., 2008). Esrrb is also capable of replacing KLF4 in somatic cell reprogramming (Feng et al., 2009). Dax1 is an orphan receptor that is important for ESC self-renewal (Niakan et al., 2006). Tcfcp2l1 colocalizes with Oct4 on many ESC promoters and may be important for optimal ESC proliferation (Chen et al., 2008b; Ivanova et al., 2006). FLAG-tagged cDNAs were stably introduced into ZHBTc4 ESCs and clones selected that express the encoded proteins at levels similar to the endogenous proteins (Figure S2A). These clones had comparable morphology and growth rate to the parental line (data not shown). Proteins were purified by our FLAG-affinity protocol, and coomassie-stained gels of the purified fractions from F-Sall4, F-Esrrb, and F-Tcfcp2l1 purifications showed prominent bands of the expected molecular weight (Figure 2A) that reacted with the FLAG antibody (Figure S2B). The presence of additional bands in the transcription factor purifications suggests the efficient copurification of associated proteins. F-Dax1 was not visible by coomassie blue staining (Figure 2A), although it was almost completely depleted from the nuclear extract by the purification (Figure S2B). Together with the weaker anti-FLAG western signals of F-Dax1 extracts and purified Dax1 fractions, compared to the other FLAG proteins (not shown), this suggests a relatively low expression level of F-Dax1 (and therefore of endogenous Dax1) in ESCs. Figures 2B–2E provide summaries of the interacting proteins of Sall4, Dax1, Tcfcp2l1, and Esrrb (complete lists of identifications and information on Mascot scores, number of identified unique peptides, and emPAI scores are shown in Tables S2–S9). To examine the Oct4 dependence of the interaction partner associations, we also performed the purifications 16 hr after doxycycline-mediated repression of Oct4, which removes essentially all Oct4 protein from ZHBTc4-derived cells (Niwa et al., 2000; van den Berg et al., 2008). Purified fractions from two FLAG purifications of cells with or without doxycycline addition were analyzed by mass spectrometry. Doxycycline addition had no consistent effect on the vast majority of the identified interactions (Tables S2–S9). Of the proteins affected by Oct4 modulation, only Esrrb was ever identified as an Oct4 interactor (Table 1). The interaction between Esrrb and Sall4 appears to be sensitive to removal of Oct4 in the F-Sall4 purifications (Tables S2 and S6). However, the mascot scores here are close to threshold, whereas in F-Esrrb purifications where Sall4 has a high Mascot and emPAI score, removal of Oct4 had no effect (Tables S5 and S9). Taken together, this suggests that the identified interactions are unlikely to be bridged by Oct4, although many of the identified proteins also interact with Oct4.

We independently verified a number of the putative interactors of F-Sall4, F-Dax1, F-Tcfcp2l1, and F-Esrrb. Immunoprecipitation of Sall4 coprecipitated Sall1 and MTA2 (Figure S3A), V5-tagged Zfp143 (Figure S3B), and F-Nac1 (Figure S3C), whereas Sall4 is present in immunoprecipitates of MTA2 (Figure S3D) and F-Nac1 (Figure S3E). GST-Dax1 pull-downs precipitated Sall4, Sall1, Oct4, Wdr5, and Esrrb (Figure S3F). V5-Tcfcp2l1 immunoprecipitation brought down Esrrb and MTA2 (Figure S3G), whereas GST-Esrrb pull-down coprecipitated MTA2, Sall4, Ep400 (Figure S3H), V5-Dax1 (Figure S3I), and F-Tcfcp2l1 (Figure S3J). MTA2 immunoprecipitation coprecipitated Esrrb (Figure S3K).

### An Oct4-Centered Interaction Network

**Figure 3:**
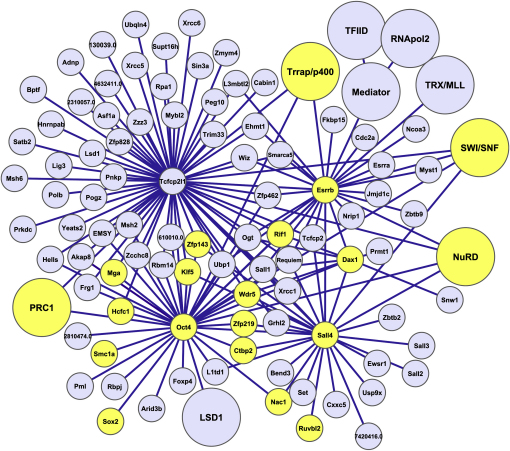
Protein Interaction Network of Oct4 and Its Associated Proteins Sall4, Dax1, Tcfcp2l1, and Esrrb The network represents the proteins present in both purifications (−Dox) of F-Sall4, F-Dax1, F-Tcfcp2l1, or F-Esrrb and/or present in F-Oct4 purifications as in Table 1 (complete lists of identifications and information on Mascot scores, number of identified unique peptides, and emPAI scores are shown in Table 1 and Tables S2–S9). Complexes are shown as larger circles. Yellow coloring indicates importance for ESC self-renewal capacity (see Table 2). See also Figure S3.

We assembled the identified interactions of Oct4, Tcfcp2l1, Dax1, Sall4, and Esrrb into an interaction network containing 166 proteins (Figure 3). This allows the visualization of the interactions between the purified tagged transcription factors and their interaction with multiple chromatin-modifying complexes (CMCs). The NuRD complex was associated with every tagged factor purified, except for Dax1 (Table 1, Figures 2B–2E). The smaller set of interactors identified for Dax1 (Figure 2C), compared to the other purified proteins, may be due to the purification of relatively small amounts of F-Dax1 protein (Figure 2A). The Mascot and emPAI scores of NuRD are highest in the F-Sall4 purifications (Figure 2B; Tables S2 and S6). Sall4 also interacts with Sall1, Sall2, and Sall3 and associates with all the other tagged factors (Figures 2B–2E). Binding of Sall4 to NuRD and Sall1 was previously observed (Yuri et al., 2009). Our data suggest that spalt proteins form a unit with NuRD, which then can associate with other transcription factors. Sall4 interactors Nac1 and Bend3 (Figure 2B) could also be part of this unit, as indicated by the fact that they were observed together in individual purifications of Tcfcp2l1 and Esrrb (data not shown). The SWI/SNF complex also associates with most tagged transcription factors (Table 1, Figures 2B, 2D, and 2E). The Trrap/p400 complex is present with relatively high mascot and emPAI scores in Esrrb and Tcfcp2l1 purifications, with many subunits detected (Figures 2D and 2E; Tables S4, S5, S8, and S9). The PRC1/Mblr complex associates, besides Oct4, also with Tcfcp2l1 (Figure 2D).

We find that the purified factors often bind efficiently to evolutionary related proteins. In addition to spalt proteins, we observed interactions between Tcfcp2l1, Tcfcp2, Ubp1, and Grhl2 (Figure 2D), all of which are related to the *Drosophila* Grainyhead transcription factors (Wilanowski et al., 2002), whereas Esrrb binds the related protein Esrra (Figure 2E). This suggests that despite diversification, these proteins can still act together in transcription regulation.

Some of the purified factors harbor extensive sets of unique interacting proteins that may mediate their specific function in ESCs. For example, Tcfcp2l1 interacts with many proteins involved in DNA metabolic processes (Figure 2D) such as DNA replication (Polb, Asf1a, Rpa1) and DNA repair (Xrcc1, 5, 6, Msh2, 6, lig3, EMSY, Prkdc, pnkp) and related pathways such as cell cycle progression or cell proliferation (Hells, Msh2, Mybl2, EMSY).

Orphan receptor Esrrb, which is related to the estrogen receptor, was found to associate with Ncoa3 and Nrip and the TRX/Mll chromatin-modifying complex (Figure 2E). Intriguingly, Esrrb also interacts with the Mediator complex, RNA polymerase II subunits (RNApol2), and TBP plus Tafs (TFIID complex; Figure 2E; Tables S5 and S9), which are all components of the basal transcription machinery (Sikorski and Buratowski, 2009). The association of Esrrb with Mediator and RNApol2 is DNA independent as shown by the fact that it was not affected by benzonase treatment of the extract (Figure 2F). Moreover, recombinant GST-Esrrb also interacted efficiently with Mediator and RNA pol2 (Figure 2G).

The network provides links with protein modification and signaling pathways. For example, Oct4 associates with Rbpj, a transcription factor that acts as the nuclear effector of the Notch signaling pathway (Bray, 2006), suggesting a connection between Notch-regulated and Oct4-regulated gene expression. Sall4 shows an interaction with Usp9x (Figure 2B), an essential component of the TGF-β/BMP signaling pathway, which activates Smad4 by removing a monoubiquitin group (Dupont et al., 2009). Another Sall4-associated factor, Cxxc5 (Figure 2B), is regulated by TGF-β signaling in neural stem cells, binds Wnt-signaling mediator Dvl, and inhibits Wnt signaling (Andersson et al., 2009). By interacting with both Usp9x and Cxxc5, spalt proteins may provide a physical link between the TGF-β and Wnt signaling pathways. Oct4, Esrrb, Tcfcp2l1, and Dax1 bind the glycosyl transferase Ogt (O-GlcNAc Transferase; Table 1, Figures 2B–2E), an enzyme that adds N-acetylglucosamine groups (O-GlcNAc) to proteins.

The network contains a number of transcription factors with a high level of interconnectivity, characteristic of network hubs. Examples of such hubs are Zfp143 and Klf5. Zfp143 interacted with Oct4, Sall4, and Tcfcp2l1 (Table 1, Figures 2B and 2D) and was present in one Esrrb purification (not shown). Klf5 was present in Oct4, Sall4, and Tcfcp2l1 purifications (Table 1, Figures 2B and 2D). The purified factors Esrrb, Tcfcp2l1, Dax1, and Sall4 were selected on their interaction with Oct4, but they also have an Oct4-independent interaction with one another. All these highly connected factors affect ESC self-renewal when depleted (Table 2), suggesting that physical interaction may play a role in regulating this process. A possible rationale for this correlation, codependent recruitment to DNA, will be tested experimentally below.

### Oct4-Dependent Recruitment of Dax1, Tcfcp2l1, and Esrrb

**Figure 4:**
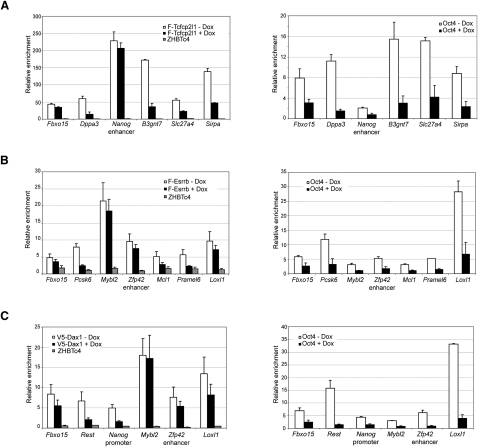
Oct4-Dependent Genome Targeting by Dax1, Tcfcp2l1, and Esrrb Left panels indicate genome binding by F-Tcfcp2l1 (A), F-Esrrb (B), and V5-Dax1 (C) at the indicated genomic regions in the absence (−Dox) or presence (+Dox) of doxycycline, as assessed by ChIP against FLAG (F-Tcfcp2l1 and F-Esrrb) or V5 (V5-Dax1) in ZHBTc4 ESCs stably expressing these tagged proteins. The ZHBTc4 parental cell line functions as a specificity control (ZHBTc4). Right panels indicate Oct4 genome binding, as assessed by Oct4 antibody ChIP, on the same regions and in the same ESCs as the corresponding left panels. Note that the addition of doxycycline diminishes expression and thereby genome binding by Oct4. Graphs show the enrichment over a control region (*Amylase*). SEM is indicated by error bars.

Our purifications showed the physical interaction of Oct4 with Dax1, Tcfcp2l1, and Esrrb. To investigate the relevance of these interactions for the ESC transcriptional network, we tested the effect of acute Oct4 depletion by 12 hr doxycycline treatment, on the recruitment of Dax1, Tcfcp2l1, and Esrrb to a number of genomic binding sites to which Oct4 also binds (Chen et al., 2008b; Kim et al., 2008). Indeed, depletion of Oct4 reduced recruitment of F-Dax1, F-Tcfcp2l1, and F-Esrrb to several of their targets (Figures 4A–4C). For example, Dax1 recruitment to the *Rest* and *Nanog* promoters, which are both also occupied by many other ESC transcription factors (Chen et al., 2008b; Kim et al., 2008), is dependent on Oct4. Our data suggest that Oct4 can provide an anchor on the DNA for the recruitment of several of its associated factors.

## Discussion

### Improved Methodology to Identify Interaction Networks in ESCs

We have improved the FLAG-affinity-based protein purification procedure by using near-physiological buffer conditions and very low detergent levels, which is possible because of our use of low-adherence plastic tubes. Previous approaches to identify interacting proteins of stem cell transcription factors used higher concentrations of detergent (Liang et al., 2008; Wang et al., 2006) and salt (Wang et al., 2006), which can cause the loss of bona fide but weak protein-protein interactions. Nonspecific elution from beads (Liang et al., 2008; Wang et al., 2006) is likely to increase background, thereby reducing the detection sensitivity and further decreasing the number of identified specific interactors.

In support of the improved sensitivity and specificity of our procedure, we identified more than 50 F-Oct4-interacting proteins by mass spectrometry (Table 1). Our increased sensitivity detected the efficient association of Oct4 with all components of NuRD. The previously claimed existence of a NuRD subcomplex with Oct4 may therefore have been the result of a limited detection efficiency (Liang et al., 2008). We subsequently applied our protocol to purify four Oct4-interacting factors, Sall4, Tcfcp2l1, Dax1, and Esrrb, and to identify their associated proteins. The combined identified interactions of the five purified factors resulted in a dense interaction network that contains more than 160 proteins. In a previous study, 35 proteins were identified in a Nanog-centered interaction network, resulting from six purified factors (Wang et al., 2006). Proteins identified in the Nanog purifications included Oct4, Dax1, Zfp281, and Nac1, but in the reverse experiment, Nanog was not identified by mass spectrometry analyses of Oct4, Dax1, Zfp281, and Nac1 purifications (Wang et al., 2006). We did not identify Nanog in our purifications of either Oct4 and Dax1. Nanog may be hard to detect by mass spectrometry, possibly because of a relative resistance to digestion into tryptic peptides.

The increased sensitivity of our procedure does not appear to come at the cost of a higher false positive rate. Three-quarter of the identified F-Oct4 interactors were also present in an endogenous Oct4 immunoprecipitation, providing a strong validation of our methodology. Further evidence of the reliability of our procedure is the reverse identification of Oct4 in all the samples of the purified transcription factors. Moreover, we independently verified 23 interactions, several of which were done in two directions, by immunoprecipitations and GST pull-downs combined with western blotting.

### Multiple Network Connections with Chromatin- and Protein-Modifying Factors

Our interaction network shows the efficient association of the purified transcription factors with several chromatin-remodeling complexes previously reported to be important for ESC self renewal (Table 2). Genome-wide analyses of binding sites in mouse ESCs have been reported for SWI/SNF (Ho et al., 2009; Kidder et al., 2009) and PRC1 (Boyer et al., 2006; Ku et al., 2008). The SWI/SNF complex binds broadly to several kilobases around the start site of many genes expressed in ESCs, including Oct4 target genes (Ho et al., 2009; Kidder et al., 2009). PRC1 also covers several kilobases around promoters enriched for both H3K27me3 and H3K4me3 and shows overlapping binding with Oct4 (Boyer et al., 2006; Endoh et al., 2008). ESC transcription factors such as Oct4, Sox2, Nanog, Esrrb, and Tcfcp2l1 often cluster more closely together (Chen et al., 2008b; Kim et al., 2008). This suggests that transcription factors may not be necessary for the continual targeting of these CMCs but recruitment may occur by initial local targeting followed by chromatin modification, thereby creating the appropriate binding surface that facilitates further spreading. CMCs often contain subunits with domains that recognize specific histone modifications (Taverna et al., 2007) and are therefore well equipped to bind specific promoter chromatin environments. Dependence both on histone marks and transcription factors would allow for multiple mechanisms of fine-tuning CMC recruitment.

Oct4, Esrrb, Tcfcp2l1, and Dax1 all bind the glycosylating enzyme Ogt, which adds O-GlcNAc groups to proteins. Recently, human Oct4 was shown to be modified by O-GlcNAc (Webster et al., 2009). O-GlcNAc modification can regulate the activity of many transcription factors (Issad and Kuo, 2008). Modification of Mll5 by Ogt was shown to be required for its histone H3K4 methylation activity and induction of granulocytic differentiation in HL60 cells (Fujiki et al., 2009). The association of Ogt with multiple ESC transcription factors suggests that the O-GlcNac modification may also regulate ESC transcriptional networks.

### Sall4, Tcfcp2l1, and Esrrb Have Unique Sets of Interacting Proteins

Some of the purified factors have extensive sets of interacting proteins that were not observed in other purifications. For example, spalt protein Sall4 is linked to TGF-β and Wnt signaling through association with Usp9x and Cxxc5, respectively. In *Drosophila* wings, *spalt* genes are regulated by TGF-β signaling, and disruption of TGF-β signaling phenocopies the effect of *spalt* mutations on wing patterning (de Celis et al., 1996). The Sall4-Usp9x association shows that spalt proteins are also connected to the TGF-β pathway by physical interaction. Tcfcp2l1 associates with several factors involved in DNA replication, DNA repair, or cell cycle regulation, suggesting that Tcfcp2l1 may link these pathways in ESCs. Tcfcp2l1 knockdown affected cell growth but no effect on self-renewal was reported (Ivanova et al., 2006). This may suggest that Tcfcp2l1 regulates cell cycle progression in ESCs and senses input from DNA replication and repair processes. Consistent with a role of Tcfcp2l1 in cell cycle regulation, Tcfcp2l1 was shown to colocalize on many promoters with transcription factor E2f1 (Chen et al., 2008b), a cell cycle regulator that binds and regulates many DNA replication and DNA repair genes (Ren et al., 2002).

An intriguing interaction is that of Esrrb with basal transcription machinery complexes Mediator, TFIID, and RNApol2, as well as with the TRX/Mll chromatin-modifying complex and Ncoa3. Mediator, TRX/Mll, and Ncoa3 also bind to the ligand-binding domain of the estrogen receptor, which is related to Esrrb, and are essential cofactors for estrogen receptor-dependent transcriptional activation in mammary cells (Kang et al., 2002; Mo et al., 2006; Shang et al., 2000). To date it is unknown how ESC transcription factor binding at promoters leads to the recruitment of the basal transcription machinery to activate transcription. By analogy to estrogen receptor in mammary cells, Esrrb may provide for such a function in ESCs.

### Interactions between ESC Transcription Factors

Our purifications identified a number of transcription factors as interaction hubs, as they interacted with many of the other transcription factors in the network. Examples of such hubs are Zfp143 and Klf5 but also the purified factors Oct4, Esrrb, Sall4, Dax1, and Tcfcp2l1 (Figure 3). Esrrb, Tcfcp2l1, and Dax1 were shown to cluster across the genome to distinct sets of Oct4 binding sites, suggesting the possibility of cooperativity. We indeed found that all three factors depend on Oct4 for efficient targeting of several of their shared binding sites with Oct4. This suggests that Oct4 DNA binding in some cases provides an anchor that, by physical interaction, facilitates the binding of other transcription factors. A paradigm for such a recruitment mechanism could be the proximal promoter of the *Nanog* gene, which contains an Oct-Sox motif 170 base pairs upstream from the transcription start site. Oct4 and Sox2 were shown to regulate *Nanog* expression by synergistic binding to this motif (Kuroda et al., 2005; Rodda et al., 2005). By using ChIP and EMSA analysis, we have recently shown that the function of the *Nanog* proximal promoter depends on the cooperative interaction between Oct4 and Esrrb (van den Berg et al., 2008). Here we show that Dax1 depends on Oct4 for its binding to the *Nanog* proximal promoter. Nac1 also binds to the *Nanog* proximal promoter (Kim et al., 2008), while binding of interaction hubs Klf5 and Zfp143 to sequences in the *Nanog* proximal promoter regulate its activity (Chen et al., 2008a; Parisi et al., 2008). In summary, at least six Oct4-associated proteins (Sox2, Esrrb, Dax1, Nac1, Klf5, and Zfp143) bind the *Nanog* proximal promoter, of which at least three do so in an Oct4-dependent manner (Sox2, Esrrb, and Dax1). Such a strong correlation could be a coincidence, but may also reflect a scenario in which multiple transcription factors bind in close proximity, depending both on DNA sequence recognition and protein-protein interactions and together ensure the appropriate *Nanog* expression level. Interestingly, a predicted consensus motif for common target genes of two sets of ESC transcription factors, including Oct4, Sox2, Dax1, Klf4, Nac1, Esrrb, and Nanog, was found to be almost identical to the Oct4-Sox2 binding site (Chen et al., 2008b; Kim et al., 2008). This suggests that a recruitment mechanism dependent on DNA sequence and protein-protein interaction, as we propose here for the *Nanog* promoter, may have many equivalents in the ESC genome.

## Experimental Procedures

### Cell Culture and DNA Constructs

Mouse ESC lines were grown on gelatin-coated dishes without feeders, as described previously (van den Berg et al., 2008). The coding sequences of Sall4, Dax1, Tcfcp2l1, and Esrrb were amplified from mouse ESC cDNA and inserted with an N-terminal double FLAG-tag (Sall4, Dax1, Esrrb), C-terminal double FLAG-tag (Tcfcp2l1), or N-terminal V5-tag (Dax1) into a pPyCAG-driven expression vector. ZHBTc4 ESCs (Niwa et al., 2000) were transfected with Lipofectamine 2000 (Invitrogen), clones were selected by 1 μg/ml puromycin, and expression of the tagged proteins in selected clones was tested by western blot analysis with FLAG (Sigma) and V5 (Invitrogen) antibodies. For transcription factor purifications from ESCs in the absence of Oct4, 1 μg/ml doxycycline (Sigma) was added for 16 hr before processing.

### Protein Purifications

FLAG-tagged transcription factor containing ZHBTc4 cells and control ZHBTc4 cells were expanded to five 14 cm diameter dishes, washed with PBS, and scraped off, and nuclear extracts were prepared (Dignam et al., 1983) and dialyzed to buffer C-100 (20 mM HEPES [pH 7.6], 0.2 mM EDTA, 1.5 mM MgCl_2_, 100 mM KCl, 20% glycerol). 60 μl of anti-FLAG M2 agarose beads (Sigma) equilibrated in buffer C-100 were added to 1.5 ml of nuclear extract in No Stick microcentrifuge tubes (Alpha Laboratories) and incubated for 3 hr at 4°C in the presence of 225 units of Benzonase (Novagen). Beads were washed five times for 5 min with buffer C-100 containing 0.02% NP-40 (C-100^∗^) and bound proteins eluted four times for 15 min at 4°C with buffer C-100^∗^ containing 0.2 mg/ml FLAG-tripeptide (Sigma). Elutions were pooled, TCA precipitated, and proteins separated by polacrylamide gel electrophoresis stained with the sensitive Colloidal Blue Staining Kit (Invitrogen) and analyzed by mass spectrometry (see Supplemental Experimental Procedures). For immunoprecipitation of endogenous Oct4 complexes, 10 μg of Oct3/4 antibody (sc-8628, Santa Cruz) or goat IgG (Santa Cruz) was cross-linked to 50 μl protein G Sepharose beads (Amersham). Antibody beads, equilibrated in C-100^∗^ and blocked with 0.1 mg/ml insulin (Sigma), 0.2 mg/ml chicken egg albumin (Sigma), and 1% fish skin gelatin (Sigma), were added to 1 ml of nuclear extracts made from 46C ESCs (Ying et al., 2003) containing Benzonase for 3 hr at 4°C in No Stick microcentrifuge tubes, washed five times for 5 min with C-100^∗^ at 4°C, and boiled in SDS-loading dye. For smaller-scale immunoprecipitations, 20 μl beads and 200 μl extract was used. The following antibodies were used: anti-Mi2β, anti-Mbd3 (kind gifts from Paul Wade), anti-Mta2 (8106, Abcam), anti-Mta1 (sc-9445, Santa Cruz), anti-Sall4 (a gift of Matthias Treir), anti-Lsd1 (ab17721, Abcam), anti-Med1 (sc-8998, Santa Cruz), and anti-RNA polymerase II (largest subunit, sc-899, Santa Cruz).

### GST Pull Down

The GST-fusion expression constructs were created by inserting mEsrrb, mDax1, or mTcfcp2l1 cDNA into pGEX-2TK. GST-fusions and GST were expressed in BL21 LysS bacteria (Invitrogen). Cells were lysed in bacterial lysis buffer (25 mM HEPES [pH 7.6], 5 mM MgCl_2_, 150 mM NaCl, 10% glycerol, 0.1% NP-40, 50 μM ZnCl_2_, protease inhibitors) and sonicated, and GST fusion proteins were bound to glutathione-sepharose beads (GE Healthcare), equilibrated in C-100^∗^, and incubated with 46C nuclear extract in No Stick tubes for 2 hr at 4°C in the presence of Benzonase. Bound proteins were analyzed by western blotting.

### Chromatin Immunoprecipitation

For ChIPs in the absence of Oct4, doxycycline was added to the cells for 12 hr before processing. 5 × 10^7^ ESCs were used per chromatin immunoprecipitation. Anti-Oct4 and anti-V5 ChIPs were performed on dual-crosslinked chromatin, as previously described (van den Berg et al., 2008). For anti-FLAG ChIP, chromatin was cross-linked for 10 min at RT with 0.4% formaldehyde. Cross-linking reactions were stopped by addition of 0.125 M glycine. ChIPs were carried out according to the online Millipore protocol; anti-FLAG and anti-V5 beads (Sigma) were preblocked with 0.5 mg/ml BSA, 0.2 mg/ml salmon sperm DNA for 3 hr at 4°C. PCR-amplified genomic regions are in Supplemental Experimental Procedures.

### Protein Interaction Network Criteria and References

Criteria for inclusion as Oct4-interacting protein in Table 1 are present in three out of four experiments (three F-Oct4 purifications and one endogenous Oct4 immunoprecipitation) with a Mascot score higher than 50 and at least 3-fold higher than the corresponding control experiment. Criteria for inclusion in Tables S2–S9 are: Present in both tagged transcription factor purifications (−Dox) with a Mascot score higher than 50 and 3-fold higher than the corresponding control experiment. In case of protein identifications with mascot score values between 50 and 60 or protein identifications based on one peptide, individual peptide MS/MS spectra were checked manually and either interpreted as valid identifications or discarded. Cytoskeletal and cytoplasmic proteins were removed from the data set. Transcription factor status and subunit composition of the complexes were assigned according to the Uniprot database. Correlation between transcription factor occupancy (Chen et al., 2008b; Kim et al., 2008) was scored as positive when >0.2. Promoter occupancy by Klf5 was assigned as overlapping with Klf4, as shown (Jiang et al., 2008). Genes bound by Oct4 were assigned according to the detection of Oct4 at their promoter (Kim et al., 2008) or ChIP sequencing data showing an association score >0.3 (Chen et al., 2008b). Microarray data on genes regulated by Oct4 (Table 2) are from Figure S4 in Sharov et al. (2008). Genes were scored as regulated by Oct4 if they showed at least 1.5-fold up- or downregulation within 48 hr after shutdown of Oct4 transcription by addition of doxycycline to ESC line ZHBTc4 and 2-fold difference within the time course of the experiment (5 days).

## Figures and Tables

**Table 1:** Oct4-Interacting Proteins as Identified by Mass Spectrometry Analysis of Purified Oct4 Samples

Protein	Accession	Flag#1		Flag#2		Flag#3		Oct4-IP		Average Mascot
Mascot^a^	Pept.^b^	Mascot^a^	Pept.^b^	Mascot^a^	Pept.^b^	Mascot^a^	Pept.^b^
Oct4 (Pou5f1)	gi|200118	532	7	987(161)	14(2)	990(233)	15(5)	909	11	854

**NuRD Complex**										

Mi2β (Chd4)	gi|39204553	2041(113)	33(4)	2576	42	2537(287)	50(7)	2959(143)	42(2)	2528
Mta1	gi|15077051	930	13	1433	22	1411	25	1235	17	1252
Gatad2a	gi|148696823	1030	14	1154	17	1575(358)	23(7)	926	12	1171
Mta2	gi|51491880	875	13	1294	19	1358	23	1003(58)	13(1)	1092
Gatad2b	gi|120577529	697	10	961	14	1346(181)	20(3)	623	7	906
Hdac1	gi|2347180	928(171)	13(3)	815(84)	12(1)	801(171)	15(4)	790(275)	9(5)	833
Mbd3	gi|7305261	563	7	525	7	793	14	1084(112)	21(1)	741
Mta3	gi|18381007	521	7	402	7	870	17	1010(69)	13(2)	700
Hdac2	gi|3023934	879(190)	11(3)	656(84)	11(1)	654	11	700(201)	9(3)	722
Rbbp7	gi|2494892	584(146)	9(4)	472	9	780(409)	14(7)	520	7	589

**SWI/SNF Complex**										

Baf155(Smarcc1)	gi|30851572	616	13	558	9	872	15	420(328)	7(7)	616
Brg1 (Smarca4)	gi|76253779	384	7	618	11	444	10	525(362)	10(8)	492

**PRC1 Complex**										

Phc1	gi|30923312	256	5	507	7	630	10	-	-	348
Ring1B (Rnf2)	gi|109157342	273	4	155	2	297(53)	6(2)	251(82)	4(1)	244
Rybp	gi|5381327	95(51)	1(1)	94	1	127	2	107	1	105

**Trrap/p400 Complex**										

Trrap	gi|124486949	154	4	273	4	134	3	496	7	264
Ep400	gi|27348237	261	6	91	1	231	5	77	2	165

**LSD1 Complex**										

Lsd1	gi|51315882	174	4	604	10	640	13	97	2	378
Zmym2	gi|28175571	189	2	533	8	296	5	-	-	254
Rcor2	gi|17298682	93	2	163	2	272	5	241	3	192

**Transcription Factors**										

Sall4	gi|81913723	2622(709)	30(10)	2526(125)	31(2)	2574(594)	38(12)	2554(636)	29(8)	2569
Sall1	gi|14164331	1987	24	2371	30	2088	31	1822	26	2067
Zfp219	gi|30794418	297	4	620	10	430	7	505	6	463
Arid3b	gi|9790033	257	3	301	4	1030	17	113	2	426
Wdr5	gi|16554627	452(68)	5(2)	222	2	468(67)	7(2)	447	5	397
Zfp462	gi|114431238	-	-	64	2	256	7	985	16	326
Sox2	gi|127140986	214	4	-	-	344	5	444	4	250
Mga	gi|6692607	-	-	375	6	348	10	247	5	242
Ubp1	gi|7305605	290	6	131	2	236	5	430	5	242
Nac1	gi|31543309	-	-	315	5	287	5	269	5	217
Hcfc1	gi|4098678	98	4	293	5	419(59)	10(1)	-	-	202
Hells	gi|12232371	-	-	316	6	287	6	53	2	164
Rbpj	gi|94400775	61	1	174	3	88	3	307	5	157
Tcfcp2l1	gi|90101766	227	4	125	2	61	2	213	4	156
Requiem	gi|6755314	304	4	150	2	-	-	157	3	153
Esrrb	gi|124375796	117	2	69	1	134	3	256	4	144
Pml	gi|9506979	136	2	66	2	333	7	-	-	134
Foxp4	gi|161016782	-	-	71	1	349	7	52	1	118
Ctbp2	gi|6753548	-	-	128	3	231	4	97	2	114
Dax1	gi|6671531	77	1	97	2	135	3	122	2	108
Zfp143	gi|22902397	186	3	-	-	118	3	84	1	97
Klf5	gi|31981873	-	-	70	1	132	2	111	1	78

**Other**										

Rif1	gi|47078460	2343	31	3370	40	2213	35	2421(1026)	31(12)	2587
L1td1	gi|148698953	271	3	311	5	497(337)	9(6)	196(58)	3(1)	319
Akap8	gi|31560394	92	1	298	4	358	6	311	4	264
Msh2	gi|30047836	298	5	142	4	522	11	54	1	254
Ogt	gi|13775066	148	2	149	4	671(160)	15(5)	-	-	242
Rbm14	gi|16307494	179	2	90	1	463(57)	9(2)	163	2	224
Frg1	gi|17376286	139	2	394	6	180	4	155	2	217
Smc1a	gi|123220915	74	3	433	10	243	8	-	-	187
Emsy	gi|124249084	144	3	104	1	429	7	-	-	170
0610010K14Rik	gi|81917220	103	2	175	2	222	3	-	-	125
2810474O19Rik	gi|148678819	69	3	69	1	213	5	-	-	88
Zcchc8	gi|148687677	97	2	84	1	106	2	-	-	72

Thresholds for inclusion of the identified proteins are in Experimental Procedures. See also Table S1.

a Mascot score for the specified protein in the Oct4 sample, purified by FLAG affinity or Oct4 immunoprecipitation (Oct4-IP). Mascot score for the specified protein in the corresponding control purification, if present, is in parentheses.

b Number of identified unique, nonredundant peptides for the specified protein in the Oct4 sample. Number of identified unique peptides in the control purification is in parentheses.

**Table 2:** Transcriptional Network and Phenotype of Oct4-Interacting Proteins

Protein	Promoter Co-occup. with Oct4^a^	Gene Bound by Oct4^a^	Expression Change upon Oct4 Depletion^b^	ESC Depletion Phenotype^c^	Developmental Phenotype^d^ (Emb. Day of Lethality)
**NuRD Complex**					

Mi2beta	-	no	no	-	-
Mta1	-	no	no	differentiation	-
Gatad2a	-	no	no	not detected	∼E10.5
Mta2	-	yes	no	not detected	-
Gatad2b	-	no	no	-	-
Hdac1	-	no	no	reduced proliferation	before E10.5
Mbd3	-	yes	no	increased self-renewal	∼E8.5
Mta3	-	yes	no	-	-
Hdac2	-	yes	no	not detected	viable
Rbbp7	-	no	down	-	-

**SWI/SNF Complex**					

Baf155	-	yes	down	differentiation	before E5.5
Brg1	-	no	no	differentiation	before E6.5

**PRC1/Mblr Complex**					

Phc1	-	yes	down	-	perinatal
Ring1b	yes	no	no	differentiation	before E10.5
Rybp	-	yes	down	not detected	before E7.5

**Trrap/p400 Complex**					

Ep400	-	no	no	differentiation	∼E9.5
Trrap	-	no	no	differentiation	∼E3.5

**LSD1 Complex**					

Lsd1	-	no	no	reduced proliferation	before E7.5
Zmym2	-	no	no	-	-
Rcor2	-	yes	down	-	-

**Transcription Factors**					

Sall4	-	yes	no	differentiation prone	before E5.5
Sall1	-	yes	no	not detected	peri-natal
Zfp219	-	yes	down	differentiation	-
Arid3b	-	no	no	-	before E11.5
Wdr5	-	no	down	differentiation	-
Zfp462	-	yes	down	-	-
Mga	-	yes	no	differentiation	-
Sox2	yes	yes	down	differentiation	before E7.5
Ubp1	-	no	no	-	∼E11.5
Nac1	yes	no	no	differentiation	viable
Hcfc1	-	no	no	differentiation	-
Hells	-	no	down	-	-
Rbpj	-	yes	no	not detected	before E10.5
Tcfcp2l1	yes	yes	down	reduced proliferation	-
Requiem	-	no	no	-	-
Esrrb	yes	yes	down	differentiation	∼E10.5
Pml	-	yes	down	-	viable
Foxp4	-	no	no	-	∼E12.5
Ctbp2	-	no	no	increased self-renewal	∼E10.5
Dax1	yes	yes	down	differentiation	-
Zfp143	yes	yes	no	differentiation	-
Klf5	yes	yes	down	differentiation	before E8.5

**Other**					

Rif1	-	yes	down	differentiation	-
L1td1	-	no	no	-	-
Akap8	-	no	no	-	-
Msh2	-	yes	no	not detected	not detected
Smc1a	-	no	no	differentiation	-
Ogt	-	yes	no	lethality	∼E5
Rbm14	-	yes	down	-	-
Frg1	-	no	no	-	-
Emsy	-	no	no	-	-
0610010K14Rik	-	no	no	-	-
281047O19Rik	-	no	no	-	-
Zcchc8	-	no	no	-	-

a Criteria and references for promoter co-occupancy with Oct4 and encoding gene bound by Oct4 are in the Experimental Procedures.

b Expression change upon Oct4 depletion in ZHBTc4 ESCs; for criteria see Experimental Procedures.

c ESC phenotype upon knockout, or knockdown by RNA interference; references in Supplemental Data.

d Developmental phenotype upon knockout; references in Supplemental Data.

## Supplemental Information

Document S1. Supplemental Experimental Procedures, Three Figures, and Nine Tables
