# Supplementary material for: An Oct4-Centered Protein Interaction Network in Embryonic Stem Cells
Source: Cell Stem Cell. 2010 Apr 2;6(4):369–81. doi: 10.1016/j.stem.2010.02.014 (PMC2860243; doi:10.1016/j.stem.2010.02.014)
Supplement: Document S1. Supplemental Experimental Procedures, Three Figures, and Nine Tables [file mmc1.pdf]

Cell Stem Cell, Volume *6*

Supplemental Information

An Oct4-Centered Protein Interaction Network

in Embryonic Stem Cells

Debbie L.C. van den Berg, Tim Snoek, Nick P. Mullin, Adam Yates, Karel Bezstarosti, Jeroen Demmers, Ian Chambers, and Raymond A. Poot

Inventory of Supplemental Information

Supplemental Experimental Procedures

References for Oct4 interaction network

Figure S1, related to Figure 1
Figure S1 contains additional data on the Oct4 purifications and Oct4 associated proteins and is linked to Figure 1 in the main article.

Figure S2, related to Figure 2
Figure S2 contains additional data on the purifications of F-Sall4, F-Dax1, F-Tcfcp2l1 and F-Esrrb and is linked to Figure 2 in the main article.

Figure S3, related to Figure 3
Figure S3 contains additional data on interacting proteins of Sall4, Dax1, Tfcfcp2l1 and Esrrb and is linked to Figure 3 in the main article.

Table S1, related to Table 1
Table S1 contains the emPAI scores of interacting proteins in all Oct4 purifications.

Tables S2 to S5, related to Figure 2
Tables S2 to S5 contain the mascot scores and numbers of unique peptides of interacting proteins in all purifications of F-Sall4, F-Dax1, F-Tcfcp2l1 and F-Dax1, respectively.

Tables S6 to S9, related to Figure 2
Tables S6 to S9 contain the emPAI scores of interacting proteins in all purifications of F-Sall4, F-Dax1, F-Tcfcp2l1 and F-Dax1, respectively.

Supplemental Experimental Procedures

Antibodies used in Figures S1, S2, S3

anti-Sox2 (Y17, Santa Cruz), anti-Sall4 (PP-PPZ 0601-00, Perseus Proteomics), anti-Oct4 (C10, Santa Cruz), anti-Dax1 (sc-841, Santa Cruz), anti-Tcfcp2l1 (ARP32606, Aviva), anti-Esrrb (PP-H6707-00, R&D systems), anti-Flag (M2, Sigma), anti-Sall1 (PP-K9814-00, R&D), anti-Wdr5 (07-706, Upstate), anti-Ep400 (A300-541a, Bethyl Laboratories), anti-Lamin B1 (C20, Santa Cruz).

Western and RT-PCR analysis of ES cell lines

Whole cell extracts of F-Oct4 ES cells, ZHBTc4 ES cells and CGR8 ES cells were analysed by western blot with the indicated antibodies. For RT-PCR analysis, RNA was purified from $5x10^6$ cells using the RNeasy protocol (Qiagen). cDNA was synthesised using 2.5µg RNA primed with random hexamers according to the Superscript First Strand Synthesis System (Invitrogen). PCRs were performed on a Roche Lightcycler by an initial denaturation at 95°C for 5 mins, followed by 45 cycles of denaturation (95°C, 5s), annealing (58°C, 10 s) and elongation (72°C, 20 s).

Primers used for RT-PCR analysis of ES cell lines

TBP FW	ggggagctgtgatgtgaagt
TBP RV	ccaggaaataattctggctca
Klf4 FW	cgggaagggagaagact
Klf RV	gacttcctcacgccaacg
Zfp42 FW	cagctcctgcacacagaaga
Zfp42 RV	actgatccgcaaacacctg
Eras FW	gcccctcatcagactgctac
Eras RV	gcagctcaaggaagaggtgt
Dax1 FW	accgtgctctttaacccaga
Dax1 RV	ccggatgtgctcagtaagg
Fgf5 FW	gtttccagtggagcccttc
Fgf5RV	gagacacagcaaatatttccaaaa
T (Brachyury) FW	cagcccacctactggctcta
T (Brachyury) RV	gagcctggggtgatggta

Superose 6 gelfiltration of ES cell nuclear extracts

200 µl ES cell nuclear extract was separated on a 25 ml Superose 6 gel filtration column (GE Healthcare) with a flow rate of 0.1 ml per minute in C-100 buffer (20 mM Hepes pH 7.6, 0.2 mM EDTA, 1.5 mM $MgCl_2$, 100 mM KCl, 20% glycerol). 0.5 ml elution fractions were TCA precipitated, separated on SDS polyacrylamide and western blots probed with anti-Oct3/4 antibody (sc-8628, Santa Cruz) or anti-Mta2 (8106, Abcam). The Superose 6 column was calibrated with gel filtration calibration standards (GE Healthcare).

Mass spectrometric analysis

1D SDS-PAGE gel lanes were cut into 2-mm slices using an automatic gel slicer and subjected to in-gel reduction with dithiothreitol, alkylation with iodoacetamide and digestion with trypsin (Promega, sequencing grade), essentially as described by (Wilm et al., 1996). Nanoflow LC-MS/MS was performed on an 1100 series capillary LC system (Agilent Technologies) coupled to an LTQ-Orbitrap mass spectrometer (Thermo) operating in positive mode and equipped with a nanospray source. Peptide mixtures were trapped on a ReproSil C18 reversed phase column (Dr Maisch GmbH; column dimensions 1.5 cm × 100 µm, packed in-house) at a flow rate of 8 µl/min. Peptide separation was performed on ReproSil C18 reversed phase column (Dr Maisch GmbH; column dimensions 15 cm × 50 µm, packed in-house) using a linear gradient from 0 to

80% B (A = 0.1 % formic acid; B = 80% (v/v) acetonitrile, 0.1 % formic acid) in 70 min and at a constant flow rate of 200 nl/min using a splitter. The column eluent was directly sprayed into the ESI source of the mass spectrometer. Mass spectra were acquired in continuum mode; fragmentation of the peptides was performed in data-dependent mode. Peak lists were automatically created from raw data files using the Mascot Distiller software (version 2.1; MatrixScience). The Mascot search algorithm (version 2.2, MatrixScience) was used for searching against the NCBInr database (release NCBInr_20090222; taxonomy: Mus musculus) or the IPI_mouse_database (release 20090924). The peptide tolerance was typically set to 10 ppm and the fragment ion tolerance to 0.8 Da. A maximum number of 2 missed cleavages by trypsin were allowed and carbamidomethylated cysteine and oxidized methionine were set as fixed and variable modifications, respectively. The Mascot score cut-off value for a positive protein hit was set to 60, based on at least two peptides. In case of protein identifications with Mascot scores between 50 and 60, or that were based on only one peptide, individual peptide MS/MS spectra were checked manually and either interpreted as valid identifications or discarded. We also show a more quantitative measure of our identified proteins, emPAI score (Ishihama et al., 2005). emPAI score incorporates the number of peptides identified per protein (spectral counts) normalized by the theoretical number of peptides. This is a superior method over just counting the number of identified peptides, because it takes account of the fact that, for the same number of molecules, larger proteins and proteins with many peptides in the preferred mass range for mass spectrometry will generate more observed peptides.

Wilm, M., Shevchenko, A., Houthaeve, T., Breit, S., Schweigerer, L., Fotsis, T., and Mann, M. (1996). Femtomole sequencing of proteins from polyacrylamide gels by nano-electrospray mass spectrometry. Nature *379*, 466-469.
Ishihama, Y., Oda, Y., Tabata, T., Sato, T., Nagasu, T., Rappsilber, J., and Mann, M. (2005). Exponentially modified protein abundance index (emPAI) for estimation of absolute protein amount in proteomics by the number of sequenced peptides per protein. Mol Cell Proteomics *4*, 1265-1272.

Immunoprecipitation with tagged proteins
Coding sequences were amplified from mouse ES cell cDNA and inserted with an C-terminal V5-tag (Tcfcp2l1 and Zfp143) or FLAG-tag (Nac1) into a pPyCAG-driven expression vector. 46C ES cells (Ying et al., 2003) were transfected with the constructs using Lipofectamine 2000 (Invitrogen), nuclear extracts were made 24 hrs after transfection and immunoprecipitations were done, as described in the Experimental Procedures.

Primers used for amplification ChIP targets

Gene	FW primer	RV primer
Fbxo15 (-0.6kb)	TCCCCCTGTAAATTCACTCA	TAGCTAGCTGGTTGGTCCAC
Rest (-3.1kb)	CTCCCCTGGACAATAGCTTC	CGTCCTTCATTTCCTCAGTG
Nanog enhancer (-5kb)	GTCCCCGCTCCTTTTCAGCACTAACCATAC	CGGTTTGAATAGGGAGGAGGGCGTCT
Nanog promoter (-0.2kb)	AAGATGAATAAAGTGAAATGAGGTAAAGC	ACTGGGAGGGAGGGAAAGC
Dppa3 (-1.7kb)	GATCCAGCTGGTCTGAGCTA	GTGCAGGGATCATAGGAGTG
Zfp42 enhancer (-14kb)	GTGTGGTGTTGAGCAGGTGT	TGACACAAAGCTTCACTACGG
Mybl2 (-2.4kb)	GACCACTCCCAGGTTTGACT	AGGAATCTGGTGACCTCCAC
Pcsk6 (+11.5kb)	ACTTGGGATCCTCCCTTCTT	ATCCTAGGCAGTGCTGGTCT
Mcl1 (+4.7kb)	CTCCCCTTGGAAGTTAACCA	GATGGCTGACTGGAGTCTCA
Pramel6 (+8.5kb)	CTCAGGGAACGCCAGTTTAT	ATGGGATCCCCACATAGAAA
Loxl1 (+33.6kb)	TGCATTGTCAAGAAACAAAGG	TTTCTGGATAGCCCATCTCC
B3gnt7 (+7.6kb)	ATCCCACTGTTACCCAGAGC	CCCTACTCCCCGGTACTACA
Slc27a4 (+17.9kb)	TAGTCTTTGGCGGCAGTTTA	CTTCCTCCTCCCATTCTTGT
Sirpa (-5.0kb)	CTGGACTCATTGTGGATTGG	TCTGGGGATCTGGTTCTACC

References for Oct4 interaction network

References for (genome-wide) chromatin immunoprecipitation data; Oct4 (Chen et al., 2008b; Endoh et al., 2008; Kim et al., 2008), Sox2 (Chen et al., 2008b; Kim et al., 2008), Esrrb (Chen et al., 2008b; van den Berg et al., 2008), Klf5 (Jiang et al., 2008; Kim et al., 2008; Parisi et al., 2008), Dax1, Nac1 (Kim et al., 2008), Tcfcp2l1 (Chen et al., 2008b), PRC1 complex (Boyer et al., 2006; Endoh et al., 2008; Ku et al., 2008). References for ES cell and developmental phenotypes (Table 2); NuRD subunits (Hendrich et al., 2001; Kaji et al., 2006; Lagger et al., 2002; Liang et al., 2008; Marino and Nusse, 2007; Zimmermann et al., 2007), SWI/SNF subunits (Bultman et al., 2000; Fazzio et al., 2008; Kim et al., 2001; Klochendler-Yeivin et al., 2000), PRC1 subunits (Endoh et al., 2008; Hu et al., 2009; Pirity et al., 2005; Takihara et al., 1997; van der Stoop et al., 2008; Voncken et al., 2003), Trrap subunits (Fazzio et al., 2008; Herceg et al., 2001; Ueda et al., 2007), Lsd1 complex (Hildebrand and Soriano, 2002; Wang et al., 2009; Wang et al., 2007), Sall4 (Elling et al., 2006; Yuri et al., 2009; Zhang et al., 2006), Sall1 (Nishinakamura et al., 2001), Zfp219 (Hu et al., 2009), Wdr5 (Ding et al., 2009), Ubp1 (Parekh et al., 2004), Mga (Hu et al., 2009), Arid3b (Takebe et al., 2006), Sox2 (Avilion et al., 2003; Masui et al., 2007), Nac1 (Mackler et al., 2008; Wang et al., 2006), Tcfcp2l1 (Ivanova et al., 2006), Rbpj (Oka et al., 1995), Esrrb (Ivanova et al., 2006; Loh et al., 2006; Luo et al., 1997), hcfc1 (Dejosez et al., 2008), Dax1 (Niakan et al., 2006), Zfp143 (Chen et al., 2008a), Pml (Wang et al., 1998), Foxp4 (Li et al., 2004), Ctbp2 (Hildebrand and Soriano, 2002; Tarleton and Lemischka, 2010), Klf5 (Parisi et al., 2008; Shindo et al., 2002), Rif1 (Loh et al., 2006), Smc1a (Hu et al., 2009), Msh2 (de Wind et al., 1995), Ogt (O'Donnell et al., 2004; Shafi et al., 2000).

REFERENCES

Avilion, A.A., Nicolis, S.K., Pevny, L.H., Perez, L., Vivian, N., and Lovell-Badge, R. (2003). Multipotent cell lineages in early mouse development depend on SOX2 function. Genes Dev *17*, 126-140.

Boyer, L.A., Plath, K., Zeitlinger, J., Brambrink, T., Medeiros, L.A., Lee, T.I., Levine, S.S., Wernig, M., Tajonar, A., Ray, M.K., *et al.* (2006). Polycomb complexes repress developmental regulators in murine embryonic stem cells. Nature *441*, 349-353.

Bultman, S., Gebuhr, T., Yee, D., La Mantia, C., Nicholson, J., Gilliam, A., Randazzo, F., Metzger, D., Chambon, P., Crabtree, G., *et al.* (2000). A Brg1 null mutation in the mouse reveals functional differences among mammalian SWI/SNF complexes. Mol Cell *6*, 1287-1295.

Chen, X., Fang, F., Liou, Y.C., and Ng, H.H. (2008a). Zfp143 regulates Nanog through modulation of Oct4 binding. Stem Cells *26*, 2759-2767.

Chen, X., Xu, H., Yuan, P., Fang, F., Huss, M., Vega, V.B., Wong, E., Orlov, Y.L., Zhang, W., Jiang, J., *et al.* (2008b). Integration of external signaling pathways with the core transcriptional network in embryonic stem cells. Cell *133*, 1106-1117.

de Wind, N., Dekker, M., Berns, A., Radman, M., and te Riele, H. (1995). Inactivation of the mouse Msh2 gene results in mismatch repair deficiency, methylation tolerance, hyperrecombination, and predisposition to cancer. Cell *82*, 321-330.

Dejosez, M., Krumenacker, J.S., Zitur, L.J., Passeri, M., Chu, L.F., Songyang, Z., Thomson, J.A., and Zwaka, T.P. (2008). Ronin is essential for embryogenesis and the pluripotency of mouse embryonic stem cells. Cell *133*, 1162-1174.

Ding, L., Paszkowski-Rogacz, M., Nitzsche, A., Slabicki, M.M., Heninger, A.K., de Vries, I., Kittler, R., Junqueira, M., Shevchenko, A., Schulz, H., *et al.* (2009). A genome-scale RNAi screen for Oct4 modulators defines a role of the Paf1 complex for embryonic stem cell identity. Cell stem cell *4*, 403-415.

Elling, U., Klasen, C., Eisenberger, T., Anlag, K., and Treier, M. (2006). Murine inner cell mass-derived lineages depend on Sall4 function. Proc Natl Acad Sci U S A *103*, 16319-16324.

Endoh, M., Endo, T.A., Endoh, T., Fujimura, Y., Ohara, O., Toyoda, T., Otte, A.P., Okano, M., Brockdorff, N., Vidal, M., *et al.* (2008). Polycomb group proteins Ring1A/B are functionally linked to the core transcriptional regulatory circuitry to maintain ES cell identity. Development *135*, 1513-1524.

Fazzio, T.G., Huff, J.T., and Panning, B. (2008). An RNAi screen of chromatin proteins identifies Tip60-p400 as a regulator of embryonic stem cell identity. Cell *134*, 162-174.

Hendrich, B., Guy, J., Ramsahoye, B., Wilson, V.A., and Bird, A. (2001). Closely related proteins MBD2 and MBD3 play distinctive but interacting roles in mouse development. Genes Dev *15*, 710-723.

Herceg, Z., Hulla, W., Gell, D., Cuenin, C., Lleonart, M., Jackson, S., and Wang, Z.Q. (2001). Disruption of Trrap causes early embryonic lethality and defects in cell cycle progression. Nat Genet *29*, 206-211.

Hildebrand, J.D., and Soriano, P. (2002). Overlapping and unique roles for C-terminal binding protein 1 (CtBP1) and CtBP2 during mouse development. Mol Cell Biol *22*, 5296-5307.

Hu, G., Kim, J., Xu, Q., Leng, Y., Orkin, S.H., and Elledge, S.J. (2009). A genome-wide RNAi screen identifies a new transcriptional module required for self-renewal. Genes Dev *23*, 837-848.

Ishihama, Y., Oda, Y., Tabata, T., Sato, T., Nagasu, T., Rappsilber, J., and Mann, M. (2005). Exponentially modified protein abundance index (emPAI) for estimation of absolute protein amount in proteomics by the number of sequenced peptides per protein. Mol Cell Proteomics *4*, 1265-1272.

Ivanova, N., Dobrin, R., Lu, R., Kotenko, I., Levorse, J., DeCoste, C., Schafer, X., Lun, Y., and Lemischka, I.R. (2006). Dissecting self-renewal in stem cells with RNA interference. Nature *442*, 533-538.

Jiang, J., Chan, Y.S., Loh, Y.H., Cai, J., Tong, G.Q., Lim, C.A., Robson, P., Zhong, S., and Ng, H.H. (2008). A core Klf circuitry regulates self-renewal of embryonic stem cells. Nat Cell Biol *10*, 353-360.

Kaji, K., Caballero, I.M., MacLeod, R., Nichols, J., Wilson, V.A., and Hendrich, B. (2006). The NuRD component Mbd3 is required for pluripotency of embryonic stem cells. Nat Cell Biol *8*, 285-292.

Kim, J., Chu, J., Shen, X., Wang, J., and Orkin, S.H. (2008). An extended transcriptional network for pluripotency of embryonic stem cells. Cell *132*, 1049-1061.

Kim, J.K., Huh, S.O., Choi, H., Lee, K.S., Shin, D., Lee, C., Nam, J.S., Kim, H., Chung, H., Lee, H.W., *et al.* (2001). Srg3, a mouse homolog of yeast SWI3, is essential for early embryogenesis and involved in brain development. Mol Cell Biol *21*, 7787-7795.

Klochendler-Yeivin, A., Fiette, L., Barra, J., Muchardt, C., Babinet, C., and Yaniv, M. (2000). The murine SNF5/INI1 chromatin remodeling factor is essential for embryonic development and tumor suppression. EMBO Rep *1*, 500-506.

Ku, M., Koche, R.P., Rheinbay, E., Mendenhall, E.M., Endoh, M., Mikkelsen, T.S., Presser, A., Nusbaum, C., Xie, X., Chi, A.S., *et al.* (2008). Genomewide analysis of PRC1 and PRC2 occupancy identifies two classes of bivalent domains. PLoS genetics *4*, e1000242.

Lagger, G., O'Carroll, D., Rembold, M., Khier, H., Tischler, J., Weitzer, G., Schuettengruber, B., Hauser, C., Brunmeir, R., Jenuwein, T., *et al.* (2002). Essential function of histone deacetylase 1 in proliferation control and CDK inhibitor repression. Embo J *21*, 2672-2681.

Li, S., Zhou, D., Lu, M.M., and Morrisey, E.E. (2004). Advanced cardiac morphogenesis does not require heart tube fusion. Science *305*, 1619-1622.

Liang, J., Wan, M., Zhang, Y., Gu, P., Xin, H., Jung, S.Y., Qin, J., Wong, J., Cooney, A.J., Liu, D., *et al.* (2008). Nanog and Oct4 associate with unique transcriptional repression complexes in embryonic stem cells. Nat Cell Biol *10*, 731-739.

Loh, Y.H., Wu, Q., Chew, J.L., Vega, V.B., Zhang, W., Chen, X., Bourque, G., George, J., Leong, B., Liu, J., *et al.* (2006). The Oct4 and Nanog transcription network regulates pluripotency in mouse embryonic stem cells. Nat Genet *38*, 431-440.

Luo, J., Sladek, R., Bader, J.A., Matthyssen, A., Rossant, J., and Giguere, V. (1997). Placental abnormalities in mouse embryos lacking the orphan nuclear receptor ERR-beta. Nature *388*, 778-782.

Mackler, S., Pacchioni, A., Degnan, R., Homan, Y., Conti, A.C., Kalivas, P., and Blendy, J.A. (2008). Requirement for the POZ/BTB protein NAC1 in acute but not chronic psychomotor stimulant response. Behavioural brain research *187*, 48-55.
Marino, S., and Nusse, R. (2007). Mutants in the mouse NuRD/Mi2 component P66alpha are embryonic lethal. PLoS ONE *2*, e519.
Masui, S., Nakatake, Y., Toyooka, Y., Shimosato, D., Yagi, R., Takahashi, K., Okochi, H., Okuda, A., Matoba, R., Sharov, A.A., *et al.* (2007). Pluripotency governed by Sox2 via regulation of Oct3/4 expression in mouse embryonic stem cells. Nat Cell Biol *9*, 625-635.
Niakan, K.K., Davis, E.C., Clipsham, R.C., Jiang, M., Dehart, D.B., Sulik, K.K., and McCabe, E.R. (2006). Novel role for the orphan nuclear receptor Dax1 in embryogenesis, different from steroidogenesis. Molecular genetics and metabolism *88*, 261-271.
Nishinakamura, R., Matsumoto, Y., Nakao, K., Nakamura, K., Sato, A., Copeland, N.G., Gilbert, D.J., Jenkins, N.A., Scully, S., Lacey, D.L., *et al.* (2001). Murine homolog of SALL1 is essential for ureteric bud invasion in kidney development. Development *128*, 3105-3115.
O'Donnell, N., Zachara, N.E., Hart, G.W., and Marth, J.D. (2004). Ogt-dependent X-chromosome-linked protein glycosylation is a requisite modification in somatic cell function and embryo viability. Mol Cell Biol *24*, 1680-1690.
Oka, C., Nakano, T., Wakeham, A., de la Pompa, J.L., Mori, C., Sakai, T., Okazaki, S., Kawaichi, M., Shiota, K., Mak, T.W., *et al.* (1995). Disruption of the mouse RBP-J kappa gene results in early embryonic death. Development *121*, 3291-3301.
Parekh, V., McEwen, A., Barbour, V., Takahashi, Y., Rehg, J.E., Jane, S.M., and Cunningham, J.M. (2004). Defective extraembryonic angiogenesis in mice lacking LBP-1a, a member of the grainyhead family of transcription factors. Mol Cell Biol *24*, 7113-7129.
Parisi, S., Passaro, F., Aloia, L., Manabe, I., Nagai, R., Pastore, L., and Russo, T. (2008). Klf5 is involved in self-renewal of mouse embryonic stem cells. J Cell Sci *121*, 2629-2634.
Pirity, M.K., Locker, J., and Schreiber-Agus, N. (2005). Rybp/DEDAF is required for early postimplantation and for central nervous system development. Mol Cell Biol *25*, 7193-7202.
Shafi, R., Iyer, S.P., Ellies, L.G., O'Donnell, N., Marek, K.W., Chui, D., Hart, G.W., and Marth, J.D. (2000). The O-GlcNAc transferase gene resides on the X chromosome and is essential for embryonic stem cell viability and mouse ontogeny. Proc Natl Acad Sci U S A *97*, 5735-5739.
Shindo, T., Manabe, I., Fukushima, Y., Tobe, K., Aizawa, K., Miyamoto, S., Kawai-Kowase, K., Moriyama, N., Imai, Y., Kawakami, H., *et al.* (2002). Kruppel-like zinc-finger transcription factor KLF5/BTEB2 is a target for angiotensin II signaling and an essential regulator of cardiovascular remodeling. Nat Med *8*, 856-863.
Takebe, A., Era, T., Okada, M., Martin Jakt, L., Kuroda, Y., and Nishikawa, S. (2006). Microarray analysis of PDGFR alpha+ populations in ES cell differentiation culture identifies genes involved in differentiation of mesoderm and mesenchyme including ARID3b that is essential for development of embryonic mesenchymal cells. Dev Biol *293*, 25-37.
Takihara, Y., Tomotsune, D., Shirai, M., Katoh-Fukui, Y., Nishii, K., Motaleb, M.A., Nomura, M., Tsuchiya, R., Fujita, Y., Shibata, Y., *et al.* (1997). Targeted disruption of the mouse homologue of the Drosophila polyhomeotic gene leads to altered anteroposterior patterning and neural crest defects. Development *124*, 3673-3682.
Tarleton, H.P., and Lemischka, I.R. (2010). Delayed differentiation in embryonic stem cells and mesodermal progenitors in the absence of CtBP2. Mech Dev *127*, 107-119.
Ueda, T., Watanabe-Fukunaga, R., Ogawa, H., Fukuyama, H., Higashi, Y., Nagata, S., and Fukunaga, R. (2007). Critical role of the p400/mDomino chromatin-remodeling ATPase in embryonic hematopoiesis. Genes Cells *12*, 581-592.
van den Berg, D.L., Zhang, W., Yates, A., Engelen, E., Takacs, K., Bezstarosti, K., Demmers, J., Chambers, I., and Poot, R.A. (2008). Estrogen-related receptor beta interacts with Oct4 to positively regulate Nanog gene expression. Mol Cell Biol *28*, 5986-5995.
van der Stoop, P., Boutsma, E.A., Hulsman, D., Noback, S., Heimerikx, M., Kerkhoven, R.M., Voncken, J.W., Wessels, L.F., and van Lohuizen, M. (2008). Ubiquitin E3 ligase Ring1b/Rnf2 of polycomb repressive complex 1 contributes to stable maintenance of mouse embryonic stem cells. PLoS ONE *3*, e2235.

Voncken, J.W., Roelen, B.A., Roefs, M., de Vries, S., Verhoeven, E., Marino, S., Deschamps, J., and van Lohuizen, M. (2003). Rnf2 (Ring1b) deficiency causes gastrulation arrest and cell cycle inhibition. Proc Natl Acad Sci U S A *100*, 2468-2473.
Wang, J., Hevi, S., Kurash, J.K., Lei, H., Gay, F., Bajko, J., Su, H., Sun, W., Chang, H., Xu, G., *et al.* (2009). The lysine demethylase LSD1 (KDM1) is required for maintenance of global DNA methylation. Nat Genet *41*, 125-129.
Wang, J., Rao, S., Chu, J., Shen, X., Levasseur, D.N., Theunissen, T.W., and Orkin, S.H. (2006). A protein interaction network for pluripotency of embryonic stem cells. Nature *444*, 364-368.
Wang, J., Scully, K., Zhu, X., Cai, L., Zhang, J., Prefontaine, G.G., Krones, A., Ohgi, K.A., Zhu, P., Garcia-Bassets, I., *et al.* (2007). Opposing LSD1 complexes function in developmental gene activation and repression programmes. Nature *446*, 882-887.
Wang, Z.G., Delva, L., Gaboli, M., Rivi, R., Giorgio, M., Cordon-Cardo, C., Grosveld, F., and Pandolfi, P.P. (1998). Role of PML in cell growth and the retinoic acid pathway. Science *279*, 1547-1551.
Wilm, M., Shevchenko, A., Houthaeve, T., Breit, S., Schweigerer, L., Fotsis, T., and Mann, M. (1996). Femtomole sequencing of proteins from polyacrylamide gels by nano-electrospray mass spectrometry. Nature *379*, 466-469.
Ying, Q.L., Stavridis, M., Griffiths, D., Li, M., and Smith, A. (2003). Conversion of embryonic stem cells into neuroectodermal precursors in adherent monoculture. Nat Biotechnol *21*, 183-186.
Yuri, S., Fujimura, S., Nimura, K., Takeda, N., Toyooka, Y., Fujimura, Y., Aburatani, H., Ura, K., Koseki, H., Niwa, H., *et al.* (2009). Sall4 is essential for stabilization, but not for pluripotency, of embryonic stem cells by repressing aberrant trophectoderm gene expression. Stem Cells *27*, 796-805.
Zhang, J., Tam, W.L., Tong, G.Q., Wu, Q., Chan, H.Y., Soh, B.S., Lou, Y., Yang, J., Ma, Y., Chai, L., *et al.* (2006). Sall4 modulates embryonic stem cell pluripotency and early embryonic development by the transcriptional regulation of Pou5f1. Nat Cell Biol *8*, 1114-1123.
Zimmermann, S., Kiefer, F., Prudenziati, M., Spiller, C., Hansen, J., Floss, T., Wurst, W., Minucci, S., and Gottlicher, M. (2007). Reduced body size and decreased intestinal tumor rates in HDAC2-mutant mice. Cancer Res *67*, 9047-9054.

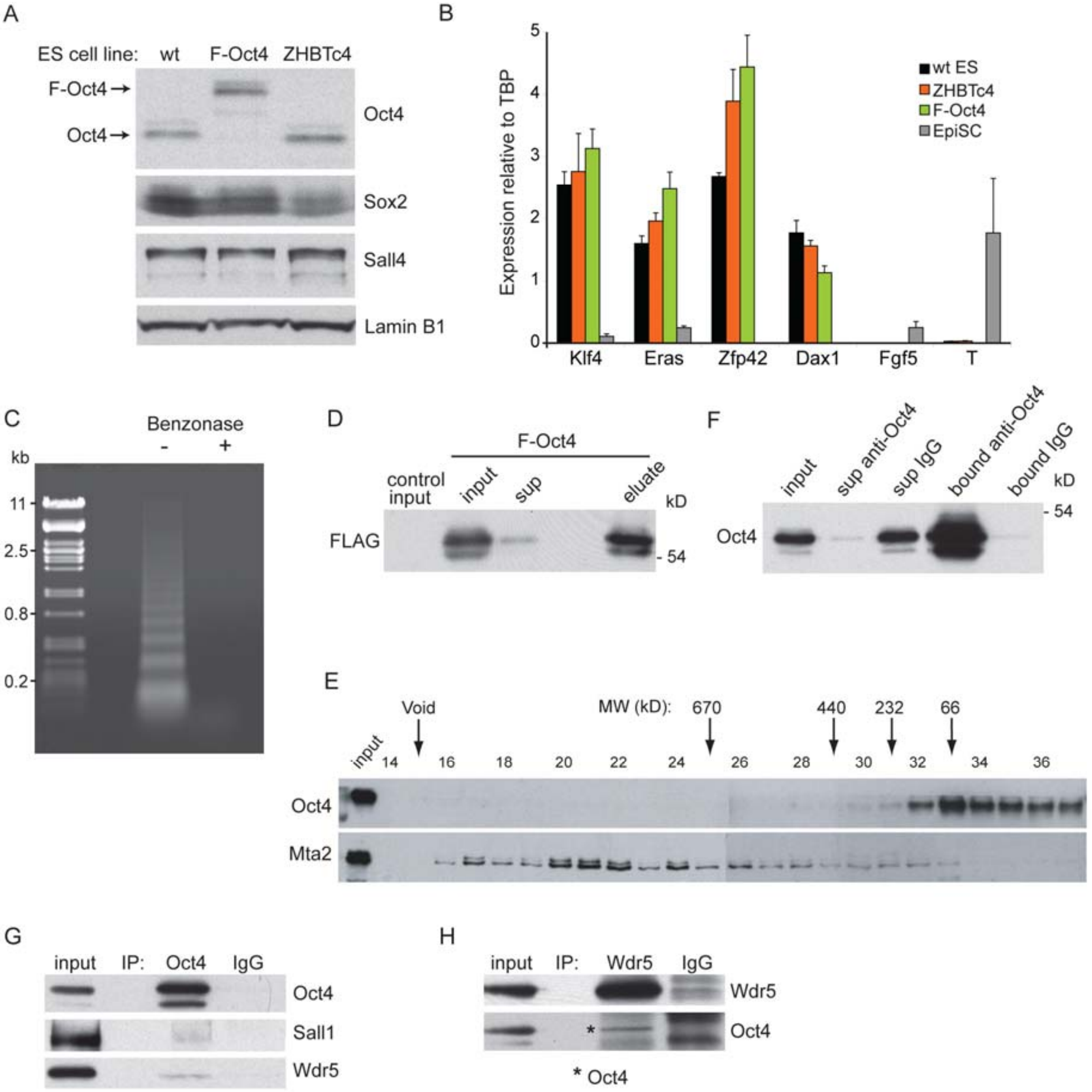

Figure S1. Additional data on Oct4 purifications

(A) Expression levels of ES cell markers in F-Oct4 and ZHBTc4 ES cells. Extracts from wild type (wt), F-Oct4 and ZHBTc4 ES cells were probed by western with the indicated antibodies. Lamin B1 served as a loading control.

(B) Quantitative RT-PCR analysis of indicated transcript levels in wild type (wt), ZHBTc4, F-Oct4 ES cells and epiblast stem cells.

(C) Treatment of ES cell nuclear extract with Benzonase removes DNA.DNA purified from 100 μl nuclear extract treated with 15 units Benzonase for 3 hrs at 4^{0}C (+ Benzonase), or not treated (- Benzonase). Size markers are indicated.

(D) F-Oct4 is depleted from nuclear extract by anti-FLAG purification, as shown by anti-FLAG western. Input, supernatant after purification (sup), eluate and control input from ZHBTc4 ES cells (not expressing F-Oct4) are indicated.

(E) Gel filtration analysis of Oct4 and NuRD subunit Mta2. Mouse ES cell nuclear extract was size-fractionated on a Superose-6 gel filtration column. Fractions were resolved on an SDS-polyacrylamide gel and probed with the indicated antibodies. Molecular weights of gel filtration standards are indicated.

(F) Oct4 is depleted from nuclear extract by anti-Oct4 immunoprecipitation. Input, supernatant (sup) and bound fraction are indicated.

(G) Verification of Oct4 interactions. Oct4 immunoprecipitates analyzed by western blots with the indicated antibodies.

(H) Wdr5 immunoprecipitates analyzed by western blots with the indicated antibodies, * indicates Oct4 band.

Figure S1, related to Figure 1

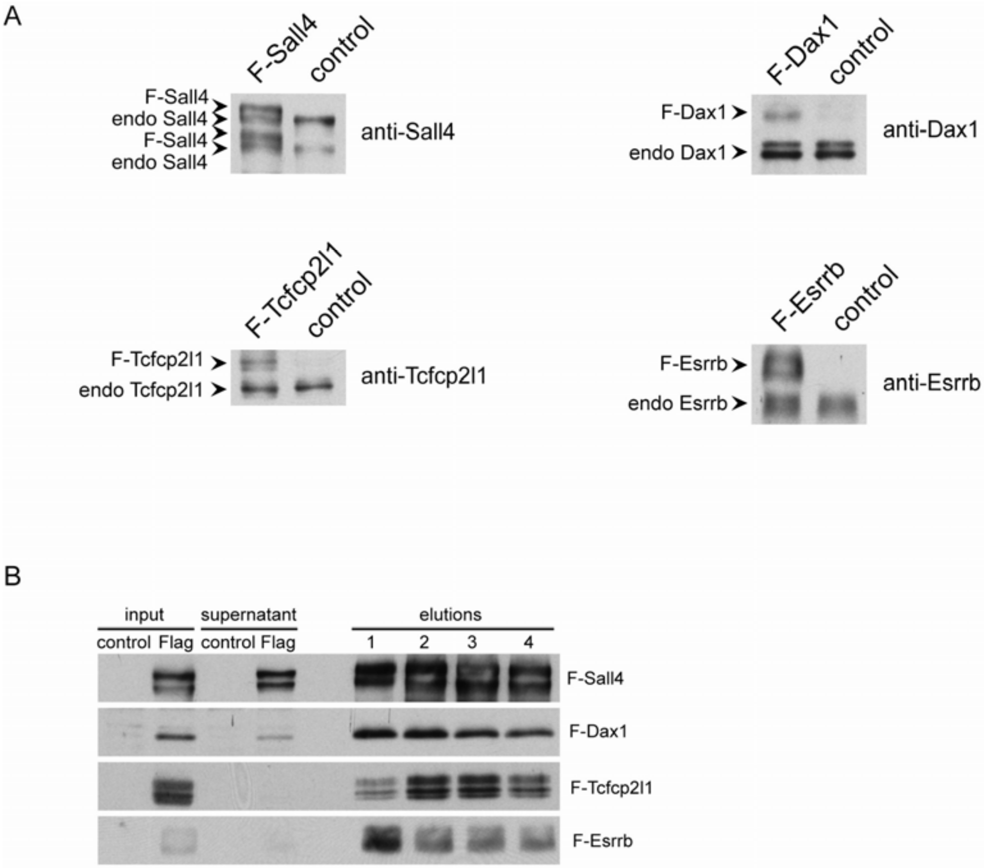

Figure S2. Additional data on the purifications of the FLAG-tagged transcription factors
(A) Expression levels of FLAG-tagged transcription factors and their endogenous counterparts in the used ES cell clones. Extracts from the ES cell clones used for FLAG-IP and FLAG-ChIP experiments were compared to control extracts from the parental ZHBTc4 ES cell line by western with the indicated antibodies. FLAG-tagged transcription factors and endogenous (endo) transcription factors are indicated by arrows.
(B) anti-FLAG western blot analysis of the purifications of FLAG-tagged Sall4 (F-Sall4), Dax1 (F-Dax1), Tcfcp2l1 (F-Tcfcp2l1) and Esrrb (F-Esrrb) from ES cell clones expressing these tagged proteins. Nuclear extracts before purification (input) and after purification (supernatant) are shown, as well as the elution fractions from anti-FLAG beads. Control extract is from the parental ZHBTc4 ES cells.

Figure S2, related to Figure 2

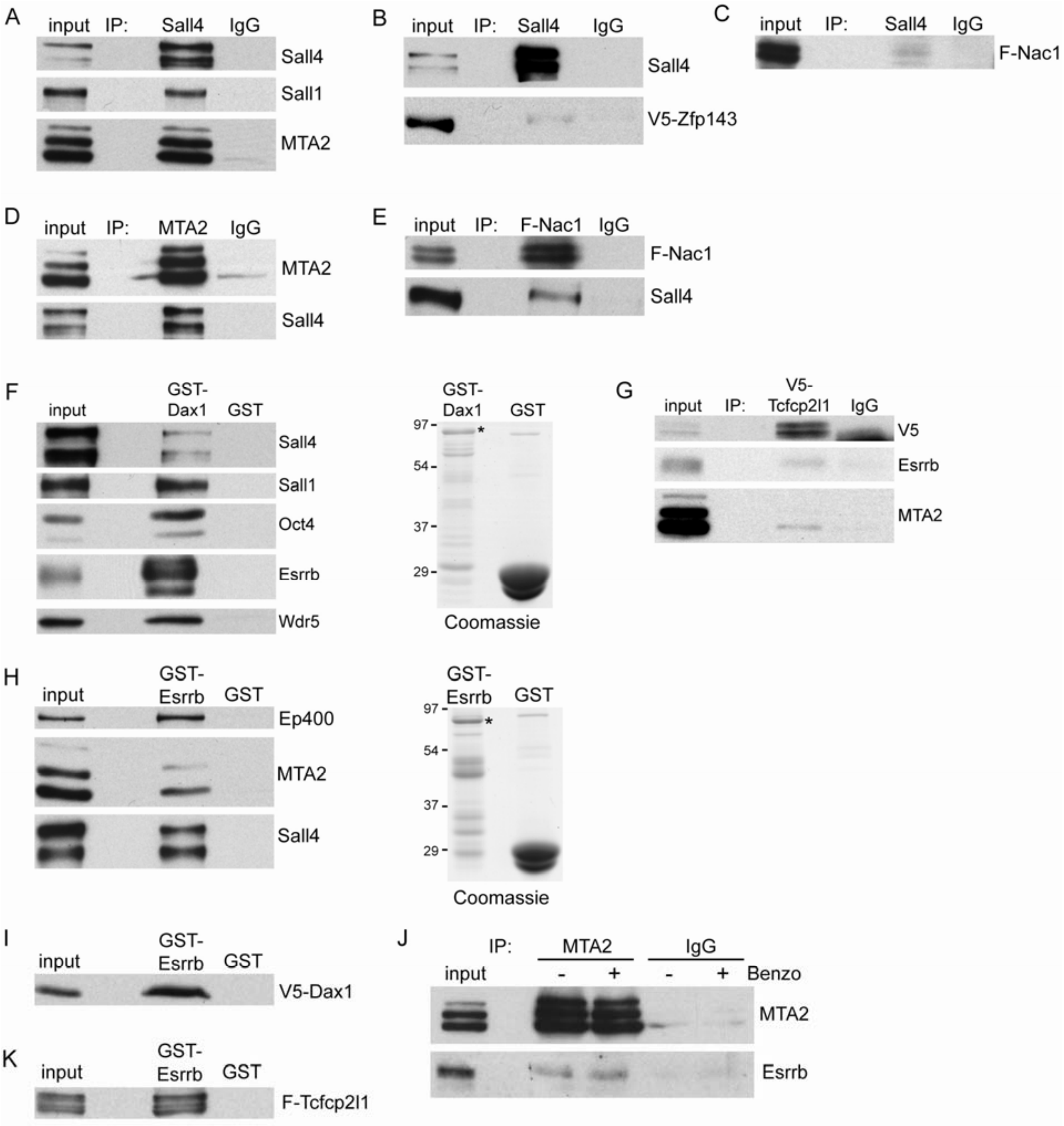

Figure S3. Verification of interactions of Sall4, Dax1, Tcfcp2l1 and Esrrb by GST-pull down and immunoprecipitation

(A) Sall4 immunoprecipitates analyzed by western blots with the indicated antibodies.

(B) Sall4 immunoprecipitates from extracts of V5-Zfp143 transfected ES cells analyzed by western blots with Sall4 and V5 antibodies.

(C) Sall4 immunoprecipitates from extracts of F-Nac1 transfected ES cells analyzed by western blot with Flag antibody.

(D) MTA2 immunoprecipitates analyzed by western blots with the indicated antibodies.

(E) F-Nac1 immunoprecipitates from extracts of F-Nac1 transfected ES cells analyzed by western blots with Flag and Sall4 antibodies.

(F, left panel) GST-Dax1 precipitates analyzed by western blots with the indicated antibodies.

(F, right panel) GST-Dax1 (*) and GST bound to beads, as used in the GST pull down, analyzed by Coomassie stained PAA gel.

(G) V5-Tcfcp2l1 immunoprecipitates analyzed by western blots with the indicated antibodies.

(H, left panel) GST-Esrrb precipitates analyzed by western blots with the indicated antibodies.

(H, right panel) GST-Esrrb (*) and GST bound to beads, as used in the GST pull down, analyzed by Coomassie stained PAA gel.

(I) GST-Esrrb precipitates analyzed by western blots with V5 antibody.

(J) GST-Esrrb precipitates analyzed by western blots with Flag antibody.

(K) MTA2 immunoprecipitates analyzed by western blots with the indicated antibodies. Benzonase was added where indicated.

Figure S3, related to Figure 3

Table S1. Oct4-interacting proteins: emPAI scores

Protein	Accession	Flag#1 emPAI [a]	Flag#2 emPAI [a]	Flag#3 emPAI [a]	Oct4-IP emPAI [a]	average emPAI [a]
**Oct4** (Pou5f1)	gi|200118	1.36	7.03(0.22)	12.38(0.32)	2.63	5.85
**NuRD complex**						
Mi2beta (Chd4)	gi|39204553	0.68(0.02)	1.38	1.57(0.12)	1.22(0.06)	1.21
Mta1	gi|15077051	0.72	1.97	2.27	0.98	1.49
Gatad2a	gi|148696823	0.9	1.48	3.47(0.45)	0.71	1.64
Mta2	gi|51491880	0.69	1.48	2.33	0.86(0.05)	1.34
Gatad2b	gi|120577529	0.73	1.16	4.16(0.18)	0.55	1.65
Hdac1	gi|2347180	2.66(0.14)	2.43(0.07)	2.36(0.21)	1.82(0.21)	2.32
Mbd3	gi|7305261	1.39	1.41	8.24	6.26(0.12)	4.33
Mta3	gi|18381007	0.44	0.45	1.96	1.21(0.05)	1.02
Hdac2	gi|3023934	2.2(0.21)	1.47(0.07)	2.55	1.32(0.21)	1.89
Rbbp7	gi|2494892	0.82(0.18)	1.09	1.66(0.82)	1.09	1.17
**SWI/SNF complex**						
Baf155(Smarcc1)	gi|30851572	0.34	0.39	0.78	0.16(0.16)	0.42
Brg1 (Smarca4)	gi|76253779	0.08	0.22	0.18	0.17(0.11)	0.16
**PRC1 complex**						
Phc1	gi|30923312	0.13	0.19	0.51	-	0.21
Ring1B (Rnf2)	gi|109157342	1.51	0.21	1.02(0.1)	0.45(0.1)	0.80
Rybp	gi|5381327	0.15(0.15)	0.15	0.3	0.15	0.19
**Trrap/p400 complex**						
Trrap	gi|124486949	0.01	0.04	0.02	0.05	0.03
Ep400	gi|27348237	0.03	0.01	0.05	0.02	0.03
**LSD1 complex**						
Lsd1	gi|51315882	0.12	0.35	0.54	0.08	0.27
Zmym2	gi|28175571	0.03	0.2	0.11	-	0.09
Rcor2	gi|17298682	0.07	0.14	0.34	0.21	0.19
**Transcription factors**						
Sall4	gi|81913723	2.78(0.29)	3.03(0.09)	6.23(0.42)	2.44(0.43)	3.62
Sall1	gi|14164331	1.05	1.58	1.8	1	1.36
Zfp219	gi|30794418	0.18	0.44	0.29	0.32	0.31
Arid3b	gi|9790033	0.19	0.27	1.7	0.13	0.57
Wdr5	gi|16554627	0.62(0.17)	0.21	1.05(0.1)	0.62	0.63
Zfp462	gi|114431238	-	0.04	0.07	0.23	0.09
Mga	gi|6692607	-	0.07	0.1	0.04	0.05
Sox2	gi|127140986	0.23	-	3.29	1.07	1.15
Ubp1	gi|7305605	0.38	0.14	0.32	0.29	0.28
Nac1	gi|31543309	-	0.37	0.34	0.37	0.27
Hcfc1	gi|4098678	0.02	0.09	0.14(0.02)	-	0.06
Hells	gi|12232371	-	0.26	0.19	0.04	0.12
Rbpj	gi|94400775	1.04	0.23	0.12	0.32	0.43
Tcfcp2l1	gi|90101766	0.3	0.14	0.06	0.14	0.16
Requiem	gi|6755314	0.49	0.17	-	0.09	0.19
Esrrb	gi|124375796	0.16	0.08	0.22	0.34	0.20
Pml	gi|9506979	0.08	0.08	0.27	-	0.11
Foxp4	gi|161016782	-	0.04	0.37	0.04	0.11
Ctbp2	gi|6753548	-	0.26	0.23	0.08	0.14
Dax1	gi|6671531	0.07	0.14	0.21	0.07	0.12
Zfp143	gi|22902397	0.05	-	0.1	0.06	0.05
Klf5	gi|31981873	-	0.07	0.14	0.07	0.07
**Other**						
Rif1	gi|47078460	0.43	0.82	0.63	0.43(0.16)	0.58
L1td1	gi|148698953	0.12	0.23	0.36(0.28)	0.13(0.04)	0.21
Akap8	gi|31560394	0.05	0.21	0.3	0.21	0.19
Msh2	gi|30047836	0.15	0.11	0.29	0.04	0.15
Smc1a	gi|123220915	0.04	0.26	0.1	-	0.10
Ogt	gi|13775066	0.06	0.1	0.58(0.06)	-	0.19
Rbm14	gi|16307494	0.12	0.05	0.55(0.05)	0.11	0.21
Frg1	gi|17376286	0.27	1.07	0.57	0.27	0.55
Emsy	gi|124249084	0.07	0.05	0.19	-	0.08
0610010K14Rik	gi|81917220	0.53	0.53	1.48	-	0.64
2810474O19Rik	gi|148678819	0.02	0.09	0.08	-	0.05
Zcchc8	gi|148687677	0.11	0.05	0.14	-	0.08

[a] emPAI score for the specified protein in the Oct4 sample. emPAI score for the specified protein in the corresponding control purification, if present, is between brackets.

Table S1, related to Table 1

Table S2. Sall4-interacting proteins as identified by mass spectrometry analysis of purified F-Sall4 samples

		Flag#1		Flag#2		Flag#3		Flag#4			
		-Dox		+Dox		-Dox		+Dox		-Dox	+Dox
Protein	Accession	Mascot[a]	Pept.[b]	Mascot[a]	Pept.[b]	Mascot[a]	Pept.[b]	Mascot[a]	Pept.[b]	Average mascot	
**Sall4**	gi|117553631	3978(70)	44(1)	3991(330)	43(4)	4530(321)	50(4)	4378(295)	48(4)	4254	4185
**NuRD complex**											
Chd4	gi|39204553	3716	54	4645	68	6021	80	5230	74	4867	4938
Gatad2a	gi|148696823	1896	21	1967	24	2055	23	2107	25	1976	2037
Mta2	gi|51491880	1717	22	1803	24	1930	26	1945	28	1824	1874
Mta1	gi|86577662	1423	20	1575	22	1727	25	1610	23	1575	1593
Gatad2b	gi|21314854	1412	16	1503	18	1598	18	1531	17	1505	1517
Mta3	gi|18381007	1336	16	1321	16	1479	19	1421	17	1408	1371
Mbd3	gi|7305261	1147	16	915	12	1112	13	1044	12	1130	980
Hdac1	gi|2347180	1144	14	1316	16	1586(200)	20(3)	1425(161)	18(2)	1365	1371
Hdac2	gi|87162464	1002	13	1055	14	1336	18	1109	15	1169	1082
Rbbp4	gi|5032027	888(120)	11(3)	934(62)	12(1)	1222(295)	15(5)	1311(241)	16(4)	1055	1123
Rbbp7	gi|157909799	709	11	967	13	1222	16	1156	15	966	1062
Mbd2	gi|7706609	477	7	371	5	601	8	386	5	539	379
**SWI/SNF complex**											
Actl6a	gi|4001805	427(128)	6(2)	606(86)	8(1)	653(211)	8(3)	628(88)	8(1)	540	617
Smarcc1	gi|30851572	239(43)	4(1)	509	10	409(81)	8(3)	304(55)	6(1)	324	407
Smarcd1	gi|576884	211	5	326	7	165	3	81	2	188	204
Arid1a	gi|124249109	158	2	129	2	92	1	-	-	125	65
Smarcb1	gi|6755578	68	1	76	1	174	2	60	1	121	68
Smarca4	gi|76253779	55	2	254	5	50	2	116	2	53	185
**Transcription factors**											
Sall1	gi|14164331	1978	26	1986	27	3094	38	2535	34	2536	2261
Bend3	gi|39841055	1585	23	1386	20	2339	30	1966	27	1962	1676
Zfp219	gi|47940209	730	9	543	6	677	9	294	4	704	419
Nac1	gi|81886163	417	6	423	6	988	12	556	8	703	490
Ruvbl2	gi|6755382	330(76)	6(1)	362(78)	5(1)	950(139)	12(2)	557(355)	8(6)	640	460
Wdr5	gi|16554627	329	4	230	4	683	10	645	11	506	437
Oct4	gi|53501	334	4	-	-	318	4	-	-	326	-
Grhl2	gi|46810275	109	2	105	2	427	7	293	6	268	199
Sall3	gi|49257163	521	7	-	-	580	7	-	-	551	-
Cxxc5	gi|19526854	132	2	230	3	287	4	449	5	210	340
Zfp143	gi|81908410	185	4	164	3	202	4	140	3	194	152
Tcfcp2	gi|15628025	82	2	-	-	277	3	163	2	180	82
Sall2	gi|49087134	145	2	157	2	164	2	172	3	155	165
Klf5	gi|31981873	90	1	105	1	194	2	-	-	142	53
Ctbp2	gi|2909779	123	3	62	1	99	2	98	2	111	80
Zbtb2	gi|85701993	76	2	-	-	141	2	54	1	109	27
Requiem	gi|6755314	106	1	208	2	109	1	-	-	108	104
Ewsr1	gi|88853581	129	2	132	2	82	1	-	-	106	66
Esrrb	gi|6166153	60	1	-	-	141	2	-	-	101	-
**Other**											
Usp9x	gi|115511018	203	2	158	2	1064	19	-	-	634	79
7420416P09Rik	gi|76253890	260	5	-	-	660	11	413	6	460	207
Set	gi|123295280	431	6	-	-	298	4	161	2	365	81
L1td1	gi|148698953	93	1	105	1	456	6	76	1	275	91

[a] Mascot score for the specified protein in the F-Sall4 sample. Mascot score for the specified protein in the corresponding control purification, if present, is between brackets.

[b] Number of identified unique, non-redundant peptides for the specified protein in the F-Sall4 sample. Number of identified unique peptides in the control purification is between brackets.

Table S2, related to Figure 2

Table S3. Dax1-interacting proteins as identified by mass spectrometry analysis of purified F-Dax1 samples

		Flag#1		Flag#2		Flag#3		Flag#4			
		-Dox		+Dox		-Dox		+Dox		-Dox	+Dox
Protein	Accession	Mascot[a]	Pept.[b]	Mascot[a]	Pept.[b]	Mascot[a]	Pept.[b]	Mascot[a]	Pept.[b]	Average mascot	
**Dax1 (Nr0b1)**	gi|6671531	928	15	860	10	736	12	711	12	832	786
**Transcription factors**											
Sall4	gi|117553631	932(245)	15(5)	646(124)	9(2)	729(204)	12(4)	894(227)	16(4)	831	770
Esrrb	gi|6166153	332	5	163	5	195	6	55	2	264	109
Snw1	gi|146149191	161	3	-	-	251	5	292	5	206	146
Sall1	gi|14164331	162	2	-	-	158	4	309	7	160	155
Wdr5	gi|16554627	130	2	90	1	161	3	65	2	146	78
Oct4	gi|200118	148	2	-	-	123	2	-	-	136	-
Prmt1	gi|30185908	57	1	-	-	55	1	80	2	56	40
**Other**											
Rif1	gi|47078460	105	2	-	-	358	7	581	11	232	291
Ogt	gi|13775066	200	3	164	3	201	4	211	4	201	188

[a, b] Equivalent to Table S2.

Table S3, related to Figure 2

Table S4. Tcfcp2l1-interacting proteins as identified by mass spectrometry analysis of purified F-Tcfcp2l1 samples

		Flag#1		Flag#2		Flag#3		Flag#4			
		-Dox		+Dox		-Dox		+Dox		-Dox	+Dox
Protein	Accession	Mascot[a]	Pept.[b]	Mascot[a]	Pept.[b]	Mascot[a]	Pept.[b]	Mascot[a]	Pept.[b]	Average mascot	
**Tcfcp2l1**	gi|134053939	2187(85)	25(1)	2133(217)	25(2)	2595	28	2234	26	2391	2184
**NuRD complex**											
Chd4	gi|39204553	2587	37	2235	35	3469	55	2487	41	3028	2361
Gatad2a	gi|30795222	1283	17	1026	14	1302	18	900	12	1293	963
Mta1	gi|15077051	909	14	937	14	1292	19	303	5	1101	620
Mta2	gi|51491880	922	13	825	12	1155	17	225	5	1039	525
Gatad2b	gi|21314854	868	10	821	9	970	11	280	3	919	551
Hdac1	gi|2347180	918(200)	13(3)	1025(161)	14(2)	728	10	492	9	823	759
Rbbp7	gi|157909799	811	10	568	9	682	12	660	12	747	614
Hdac2	gi|3023934	868	13	742	11	554	9	391	7	711	567
Mta3	gi|18381007	850	13	470	8	480	8	114	2	665	292
Mbd3	gi|7305261	498	6	585	8	641	8	447	6	570	516
Mbd2	gi|7706609	97	1	-	-	64	1	-	-	81	-
**Trrap/p400 complex**											
Trrap	gi|124486949	1741	28	1952	31	2474	44	1779	30	2108	1866
Ruvbl2	gi|6755382	1062(139)	13(2)	1196(355)	15(6)	1568(76)	19(1)	1513(78)	17(1)	1315	1355
Ep400	gi|27348237	1367	17	1158	14	1051	14	883	12	1209	1021
Ruvbl1	gi|9790083	738(128)	11(2)	830(202)	12(3)	1123(257)	15(4)	732(206)	10(3)	931	781
Actl6a	gi|4001805	679(221)	9(3)	651(88)	8(1)	479(128)	6(2)	630(86)	8(1)	579	641
Yeats4	gi|13386064	317	5	328	5	392	7	357	6	355	343
Vps72	gi|83921607	387	5	85	2	134	3	169	3	261	127
Dmap1	gi|12963557	239	4	437	6	241	3	253	4	240	345
Brd8	gi|19344050	124	2	99	2	167	3	-	-	146	50
Ing3*	gi|17390799	87	1	-	-	73	1	78	1	80	39
1600027Rik	gi|110625965	68	1	-	-	192	2	177	2	130	89
**SWI/SNF complex**											
Smarcc1	gi|30851572	1748(81)	24(3)	1523(55)	23(1)	1786(43)	26(1)	632	11	1767	1078
Smarca4	gi|76253779	749	13	753	12	1508	23	724	11	1129	739
Arid1a	gi|124249109	794	12	791	13	989	15	720	12	892	756
Actl6a	gi|4001805	679(221)	9(3)	651(88)	8(1)	479(128)	6(2)	630(86)	8(1)	579	641
Smarcc2	gi|38565930	504	8	-	-	647	9	-	-	576	-
Smarce1	gi|10181166	322(76)	5(1)	310	5	361	7	297	4	342	304
Smarcd1	gi|1549249	250	5	369	8	434	7	314	4	342	342
Smarcb1	gi|6755578	236	3	263	3	379	5	237	5	308	250
Smarcd2	gi|57013098	68	1	70	2	140	3	69	2	104	70
**PRC1/mblr complex**											
Rnf2	gi|33563274	247	4	150	3	157	3	260	4	202	205
Phc1	gi|1490546	176	3	105	3	193	2	93	1	185	99
Mblr (Pcgf6)	gi|28076973	106	2	96	2	87	2	87	2	97	92
Rybp	gi|5381327	85	1	216	3	92	1	-	-	89	108
**Transcription factors**											
Sall4	gi|81913723	2689(321)	35(4)	2252(295)	30(4)	2851(70)	34(1)	1564(330)	22(4)	2770	1908
Ubp1	gi|134032032	1775	22	2081	23	2068	24	1759	22	1922	1920
Tcfcp2	gi|15628025	868	11	1113	13	1185	13	941	12	1027	1027
Lsd1	gi|51315882	728	9	667	11	1035	15	109	3	882	388
Sall1	gi|14164331	994	12	967	10	748	10	421	7	871	694
Esrrb	gi|28277057	966	14	1451	18	610	9	868	12	788	1160
Smarca5	gi|14028669	670	12	461	8	482	10	56	1	576	259
Wdr5	gi|16554627	660	9	740	10	464	6	567	7	562	653
Peg10	gi|98985814	631(71)	8(1)	688	9	466	6	397	5	549	543
Yeats2	gi|84794613	616	9	234	4	402	6	-	-	509	117
Hcfc1	gi|34328130	323	5	379	6	679	10	105	2	501	242
Zmym4	gi|167555112	350	5	465	7	600	10	156	3	475	311
Hells	gi|12232371	345	4	229	3	522	7	150	2	434	190
Mga	gi|120444914	359	7	258	7	440	7	59	2	400	159
Zfp462	gi|85740499	711	12	692	13	85	2	56	2	398	374
Oct4	gi|125490392	375	5	-	-	321	4	-	-	348	-
Requiem	gi|6648956	332	4	295	3	362	4	197	2	347	246
Pogz	gi|111598687	496	6	452	6	90	1	-	-	293	226
Zfp143	gi|121247390	320	4	198	4	254	5	138	3	287	168
Sin3a	gi|726286	334	4	421	5	156	4	-	-	245	211
Hnrnpab	gi|6754222	300	4	251	3	171	2	401	6	236	326
Wiz	gi|46909565	208	3	91	2	262	5	64	1	235	78
Adnp	gi|55930867	268	4	198	3	199	3	51	1	234	125
Satb2	gi|20982839	220	5	100	2	246	5	162	3	233	131
Klf5	gi|31981873	209	3	151	2	232	3	84	1	221	118
Trim33	gi|119637828	98	1	344	5	311	4	97	1	205	221
Mybl2	gi|6678974	210	3	158	3	175	3	126	2	193	142
Bptf	gi|123241372	217	3	136	2	174	3	-	-	196	68
Grhl2	gi|46810275	218	4	-	-	120	2	-	-	169	-
L3mbtl2	gi|27734414	209	3	67	2	78	1	-	-	144	34
Zzz3*	gi|47847456	78	1	59	1	112	1	-	-	95	30
Ehmt1	gi|34784556	54	1	-	-	81	1	-	-	68	-
Zfp828	gi|32469497	51	2	-	-	75	2	-	-	63	-
**Other**											
Rif1	gi|47078460	1930	25	1833	26	2238	29	1403	24	2084	1618
Zcchc8	gi|169808385	822	10	780	9	747	9	-	-	785	390
Supt16h	gi|15637171	465	7	430	7	577	9	242	5	521	336
Lig3	gi|3913496	426	8	423	9	535	9	247	4	481	335
Ogt	gi|27499606	424	7	292	7	497	8	-	-	461	146
Xrcc6	gi|145587104	416	5	411	5	430	7	-	-	423	206
Xrcc5	gi|22137748	265	4	506	8	417	6	120	2	341	313
C130039O16Rik	gi|148670819	430	6	398	4	224	3	171	2	327	285
Msh2	gi|726086	260	5	251	5	393	8	-	-	327	126
L1td1	gi|124487095	378	6	520	8	216	3	74	2	297	297
Msh6	gi|6754744	448	5	101	1	134	3	-	-	291	51
Rbm14	gi|86262142	199	3	188	4	356	4	-	-	278	94
Xrcc1	gi|55391482	212	3	65	2	273	5	64	2	243	65
Polb	gi|21729749	294	5	259	4	166	3	154	3	230	207
EMSY	gi|124249084	280	3	286	4	155	3	296	4	218	291
Rpa1	gi|18390321	146	3	-	-	226	3	-	-	186	-
Prkdc	gi|124517706	119	4	-	-	198	4	-	-	159	-
4632411B12Rik	gi|37360322	176	2	-	-	82	1	-	-	129	-
Akap8	gi|5931618	149	2	178	2	107	1	-	-	128	89
Pnkp	gi|7108591	143	2	-	-	99	2	66	1	121	33
Asf1a*	gi|13384964	101	1	101	1	101	1	101	1	101	101
Cabin1	gi|70995287	135	3	-	-	62	1	-	-	99	-
2310057J16Rik	gi|61213696	78	1	104	3	55	1	-	-	67	52
Ubqln4*	gi|15805016	72	1	75	1	63	1	-	-	68	38

[a], [b] Equivalent to Table S2. * Identification by one unique peptide.

Table S4, related to Figure 2

Table S5. Esrrb-interacting proteins as identified by mass spectrometry analysis of purified F-Esrrb samples

		Flag#1		Flag#2		Flag#3		Flag#4			
		-Dox		+Dox		-Dox		+Dox		-Dox	+Dox
Protein	Accession	Mascot[a]	Pept.[b]	Mascot[a]	Pept.[b]	Mascot[a]	Pept.[b]	Mascot[a]	Pept.[b]	Average mascot	
**Esrrb**	gi|6166153	2156	25	2452	28	2005	27	1945	24	2081	2199
**SWI/SNF complex**											
Smarcc1	gi|30851572	1212(95)	20(2)	992(107)	16(3)	1048	22	568	12	1130	780
Arid1a	gi|124249109	258(51)	4	418	7	593	14	144	4	426	281
Smarca4	gi|76253779	952	16	623	12	743	15	508	10	848	566
Smarcd1	gi|1549249	573	10	310	5	451	9	362	8	512	336
Pbrm1	gi|116284015	93	1	122	2	392	9	144	3	243	133
**Trrap/p400 complex**											
Trrap	gi|124486949	1452	24	1241	22	2176	49	-	-	1814	621
Ep400	gi|27348237	828	14	778	13	1159	21	62	2	994	420
Yeats4	gi|13386064	308	6	411	8	109	3	-	-	209	206
Dmap1	gi|12963557	452	5	314	4	339	7	55	1	396	185
Brd8	gi|19344050	323	5	-	-	201	5	66	2	262	33
Ing3	gi|17390799	127	2	110	2	100	3	-	-	114	55
1600027Rik	gi|110625965	81	1	71	1	69	1	-	-	75	36
**NuRD complex**											
Chd4	gi|39204553	1332	23	714	13	653	17	327	9	993	521
Gatad2a	gi|148696823	585	8	538	7	638	11	556	10	611	547
Mta2	gi|51491880	493	9	573	9	515	10	298	6	504	436
Mta1	gi|86577662	461	6	258	4	325	7	130	3	393	194
Gatad2b	gi|21314854	464	6	538	7	416	7	241	4	440	390
Mta3	gi|18381007	128	2	312	5	172	3	-	-	150	156
Mbd3	gi|7305261	252	4	402	5	210	4	-	-	231	201
Hdac1	gi|2347180	722(198)	13(3)	791(95)	14(2)	514(98)	11(2)	488	8	618	640
Hdac2	gi|87162464	391	8	402	8	505(98)	10(2)	333	6	448	368
Rbbp4	gi|5032027	464(145)	7(3)	510(204)	7(3)	473(152)	6(2)	317(251)	5(4)	469	414
**Mediator complex**											
Med14	gi|115270972	1332	24	2113	36	1518	29	1294	24	1425	1704
Med12	gi|123226656	1092	19	1577	24	1538	28	995	20	1315	1286
Med23	gi|61651678	853	14	1239	26	1252	25	980	19	1052	1110
Med24	gi|119220579	1081	16	1024	16	867	15	675	11	974	850
Med17	gi|21450345	1040	18	1290	17	845	15	801	15	942	1046
Med1	gi|14193713	708	12	976	18	1150	23	663	15	929	820
Med16	gi|148699683	867	13	809	13	692	13	673	13	779	741
Med15	gi|32451779	798	12	805	13	615	13	465	10	706	635
Med13	gi|124286862	389	7	465	7	668	12	224	5	528	345
Med27	gi|16741439	785	12	739	13	376	7	343	5	580	541
Med25	gi|47940179	466	6	479	8	322	5	329	5	394	404
Med13l	gi|49257394	301	5	279	5	395	10	185	6	348	232
Med4	gi|13385626	285	4	733	10	405	7	326	6	345	530
Med26	gi|28466971	334	7	518	9	321	8	69	2	327	294
Med6	gi|27754027	384	6	429	6	260	6	346	8	322	388
Med8	gi|29366816	290	5	370	6	191	4	164	4	240	267
Cncc	gi|38382739	181	4	312	6	270	4	133	4	226	223
Med30	gi|19882231	274	5	209	3	114	2	78	1	194	144
Med7	gi|157266302	244	4	216	7	101	2	67	1	172	142
Med29	gi|27754101	203	4	185	3	135	2	137	3	169	161
Cdk8	gi|31652272	104	2	99	2	108	2	155	4	106	127
Med18	gi|21313064	195	4	257	5	54	1	149	3	124	203
Med19	gi|28277157	88	2	348	7	57	1	-	-	72	174
**RNApol2 complex**											
Polr2a	gi|2145091	797	13	775	13	813	18	550	11	805	663
Polr2b	gi|24418911	498	9	548	9	792	16	459	10	645	504
Polr2c	gi|29336059	334	5	454	6	214	3	91	2	274	273
Polr2g	gi|4505947	109	1	307	5	191	3	86	2	150	197
**TFIID complex**											
Taf9	gi|28175808	162	3	410	8	352	8	68	2	257	239
Taf6	gi|6678215	162	4	140	3	270	5	60	1	216	100
Taf4a	gi|123288532	109	2	71	1	270	5	146	2	190	109
Taf10*	gi|46518499	147	1	129	1	113	1	-	-	130	65
Tbp*	gi|29477183	57	1	47	1	66	1	-	-	62	24
**TRX/MLL complex**											
Wdr5	gi|16554627	414	6	475	7	275	6	244	5	345	360
Hcfc1	gi|34328130	263	5	-	-	414	7	152	3	339	76
Mll2	gi|149266757	117	2	116	2	338	8	-	-	228	58
Mll3	gi|37999865	73	2	96	2	272	7	-	-	173	48
Ashl2	gi|4009338	89	2	130	2	184	5	-	-	137	65
Rbbp5	gi|34784634	50	1	68	1	126	3	-	-	88	34
**Transcription factors**											
Sall4	gi|117553631	1329(204)	19(4)	1104(227)	17(4)	965(245)	15(5)	861(124)	14(2)	1147	983
Dax1	gi|6671531	882	12	644	9	502	9	283	6	692	464
Tcfcp2l1	gi|90101766	768	12	524	8	566	11	390	7	667	457
Fkbp15	gi|38614309	602	9	774	11	656	10	235	4	629	505
Esrra	gi|112293262	436	7	874	12	540	10	265	6	488	570
Ncoa3	gi|118026946	294	5	122	2	549	11	-	-	421	61
Ubp1	gi|134032032	480	8	587	9	336	7	46	1	408	317
Nrip1	gi|27734110	385	7	888	15	430	10	528	11	408	708
Sall1	gi|14164331	360	5	267	4	438	11	272	6	399	270
Zfp462	gi|148670321	189	5	82	3	507	12	-	-	348	41
Cdc2a	gi|13542826	278	6	360	7	313	6	71	1	295	216
Zbtb9	gi|54400753	205	5	210	4	353	6	101	3	279	156
Smarca5	gi|14028669	144	3	100	2	373	9	296	6	258	198
Wiz	gi|46909565	106	1	60	1	266	6	98	3	186	79
Requiem	gi|6648956	178	2	261	3	184	4	69	1	181	165
Jmjd1c	gi|149260924	115	2	49	1	204	4	-	-	159	25
Tcfcp2	gi|15628025	154	2	176	2	125	3	-	-	140	88
L3mbtl2	gi|27734414	63	1	-	-	217	3	73	1	140	37
Oct4	gi|125490392	107	1	-	-	158	3	-	-	133	-
Myst1	gi|21312790	64	1	71	1	138	3	-	-	101	36
Ehmt1	gi|34784556	85	1	67	1	90	3	-	-	88	34
**Other**											
Rif1	gi|47078460	1930	25	1370	20	2238	29	1656	27	2084	1513
Ogt	gi|27499606	424	7	292	7	497	8	214	5	461	253

[a, b] Equivalent to Table S2. * Identification by one unique peptide.

Table S5, related to Figure 2

Table S6. Sall4-interacting proteins: emPAI scores

		Flag#1	Flag#2	Flag#3	Flag#4		
		-Dox	+Dox	-Dox	+Dox	-Dox	+Dox
Protein	Accession	emPAI [a]	emPAI [a]	emPAI [a]	emPAI [a]	Average emPAI	
**Sall4**	gi|117553631	20.62(0.04)	17.72(0.18)	32.85(0.18)	20.65(0.13)	26.74	19.18
**NuRD complex**							
Chd4	gi|39204553	1.78	3.24	7.52	4.6	4.65	3.92
Gatad2a	gi|148696823	5.02	4.74	5.03	6	5.03	5.37
Mta2	gi|51491880	2.99	3.18	3.37	3.57	3.18	3.38
Mta1	gi|86577662	1.64	2.28	2.89	2.57	2.27	2.43
Gatad2b	gi|21314854	2.43	2.62	3.68	3	3.06	2.81
Mta3	gi|18381007	2.51	2.04	3.6	3.34	3.06	2.69
Mbd3	gi|7305261	9.72	4.22	8.69	6.1	9.21	5.16
Hdac1	gi|2347180	5.54	7.88	18.54(0.2)	8.42(0.2)	12.04	8.15
Hdac2	gi|87162464	3.52	3.81	8.93	5.12	6.23	4.47
Rbbp4	gi|5032027	1.63(0.21)	1.64(0.07)	2.2(0.29)	3.42(0.3)	1.92	2.53
Rbbp7	gi|157909799	1.66	1.86	3.67	4.37	2.67	3.12
Mbd2	gi|7706609	0.58	0.36	1.15	0.47	0.87	0.42
**SWI/SNF complex**							
Actl6a	gi|4001805	0.42(0.14)	0.75(0.07)	1.02(0.23)	0.88(0.07)	0.72	0.82
Smarcc1	gi|30851572	0.09(0.03)	0.29	0.18(0.04)	0.15(0.03)	0.14	0.22
Smarcd1	gi|576884	0.29	0.47	0.21	0.14	0.25	0.31
Arid1a	gi|124249109	0.03	0.03	0.02	0.02	0.03	0.03
Smarcb1	gi|6755578	0.08	0.08	0.16	0.08	0.12	0.08
Smarca4	gi|76253779	0.02	0.08	0.02	0.04	0.02	0.06
**Transcription factors**							
Sall1	gi|14164331	1.05	1.15	2.2	1.6	1.62	1.38
Bend3	gi|39841055	1.18	1.11	3.3	2.23	2.24	1.67
Zfp219	gi|47940209	0.6	0.37	0.52	0.23	0.56	0.3
Nac1	gi|81886163	0.34	0.42	1.03	0.6	0.69	0.51
Ruvbl2	gi|6755382	0.48(0.07)	0.39(0.07)	1.2(0.14)	0.69	0.84	0.54
Wdr5	gi|16554627	0.57	0.57	1.46	1.46	1.02	1.02
Oct4	gi|53501	0.45	-	0.45	-	0.45	-
Grhl2	gi|46810275	0.1	0.1	0.33	0.37	0.22	0.24
Sall3	gi|49257163	0.26	-	0.23	-	0.25	-
Cxxc5	gi|19526854	0.22	0.35	0.65	0.85	0.44	0.6
Zfp143	gi|81908410	0.16	0.16	0.32	0.22	0.24	0.19
Tcfcp2	gi|15628025	0.06	-	0.19	0.12	0.13	0.06
Sall2	gi|49087134	0.13	0.1	0.1	0.1	0.12	0.1
Klf5	gi|31981873	0.07	0.07	0.14	-	0.11	0.04
Ctbp2	gi|2909779	0.16	0.08	0.16	0.16	0.16	0.12
Zbtb2	gi|85701993	0.12	-	0.12	0.06	0.12	0.03
Requiem	gi|6755314	0.08	0.17	0.08	-	0.08	0.09
Ewsr1	gi|88853581	0.1	0.1	0.05	-	0.08	0.05
Esrrb	gi|6166153	0.07	-	0.15	-	0.11	-
**Other**							
Usp9x	gi|115511018	0.02	0.02	0.23	-	0.13	0.01
7420416P09Rik	gi|76253890	0.13	-	0.38	0.27	0.26	0.14
Set	gi|123295280	0.65	-	0.37	0.23	0.51	0.12
L1td1	gi|148698953	0.04	0.04	0.2	0.04	0.12	0.04

[a] emPAI score for the specified protein in the F-Sall4 sample. emPAI score for the specified protein in the corresponding control purification, if present, is between brackets.

Table S7. Dax1-interacting proteins: emPAI scores

		Flag#1	Flag#2	Flag#3	Flag#4		
		-Dox	+Dox	-Dox	+Dox	-Dox	+Dox
Protein	Accession	emPAI [a]	emPAI [a]	emPAI [a]	emPAI [a]	Average emPAI	
**Dax1 (Nr0b1)**	gi|6671531	1.12	1.26	1.56	1.57	1.34	1.42
**Transcription factors**							
Sall4	gi|117553631	0.56(0.18)	0.52(0.09)	0.43(0.13)	0.61(0.13)	0.5	0.57
Esrrb	gi|6166153	0.51	0.15	0.51	0.07	0.51	0.11
Snw1	gi|146149191	0.12	-	0.25	0.25	0.19	0.13
Sall1	gi|14164331	0.05	-	0.07	0.18	0.06	0.09
Wdr5	gi|16554627	0.34	0.16	0.56	0.35	0.45	0.26
Oct4	gi|200118	0.2	-	0.2	-	0.2	-
Prmt1	gi|30185908	0.08	-	0.08	0.08	0.08	0.04
Rif1	gi|47078460	0.03	-	0.08	0.14	0.06	0.07
Ogt	gi|13775066	0.09	0.09	0.12	0.09	0.11	0.09

[a] equivalent to Table S6.

Table S7, related to Figure 2

Table S8. Tcfcp2l1-interacting proteins: emPAI scores

		Flag#1	Flag#2	Flag#3	Flag#4		
		-Dox	+Dox	-Dox	+Dox	-Dox	+Dox
Protein	Accession	emPAI [a]	emPAI [a]	emPAI [a]	emPAI [a]	Average emPAI	
**Tcfcp2l1**	gi|134053939	13.57(0.06)	14.48(0.2)	19.04	14.53	16.31	14.51
**NuRD complex**							
Chd4	gi|39204553	0.89	0.95	1.7	0.95	1.30	0.95
Gatad2a	gi|30795222	1.71	1.11	1.58	0.91	1.65	1.01
Mta1	gi|15077051	0.89	0.89	1.24	0.24	1.07	0.57
Mta2	gi|51491880	0.63	0.63	1.14	0.14	0.89	0.39
Gatad2b	gi|21314854	1.16	0.76	1.06	0.17	1.11	0.47
Hdac1	gi|2347180	2.56(0.2)	2.79(0.2)	1.49	0.83	2.03	1.81
Rbbp7	gi|157909799	1.31	1.15	1.71	1.32	1.51	1.24
Hdac2	gi|3023934	2.14	1.33	1.19	0.72	1.67	1.03
Mta3	gi|18381007	0.87	0.35	0.42	0.1	0.65	0.23
Mbd3	gi|7305261	1.28	1.28	1.81	0.86	1.55	1.07
Mbd2	gi|7706609	0.14	-	0.08	-	0.11	-
**Trrap/p400 complex**							
Trrap	gi|124486949	0.22	0.23	0.33	0.24	0.28	0.24
Ruvbl2	gi|6755382	1.86(0.14)	2.05(0.39)	3.54(0.07)	3.24(0.07)	2.70	2.65
Ep400	gi|27348237	0.19	0.15	0.15	0.12	0.17	0.14
Ruvbl1	gi|9790083	1.38(0.14)	2.31(0.22)	2.33(0.22)	0.82(0.22)	1.86	1.57
Actl6a	gi|4001805	1.16(0.23)	1.32(0.07)	0.64(0.14)	1.16(0.07)	0.90	1.24
Yeats4	gi|13386064	1.23	1.61	1.13	0.87	1.18	1.24
Vps72	gi|83921607	0.51	0.18	0.28	0.28	0.40	0.23
Dmap1	gi|12963557	0.29	0.37	0.21	0.29	0.25	0.33
Brd8	gi|19344050	0.07	0.03	0.07	-	0.07	0.02
Ing3	gi|17390799	0.08	-	0.08	0.08	0.08	0.04
1600027Rik	gi|110625965	0.15	-	0.34	0.34	0.25	0.17
**SWI/SNF complex**							
Smarcc1	gi|30851572	1.39(0.04)	1.32(0.03)	1.46(0.03)	0.31	1.43	0.82
Smarca4	gi|76253779	0.23	0.23	0.51	0.23	0.37	0.23
Arid1a	gi|124249109	0.18	0.17	0.23	0.17	0.21	0.17
Actl6a	gi|4001805	1.16(0.23)	1.32(0.07)	0.64(0.14)	1.16(0.07)	0.90	1.24
Smarcc2	gi|38565930	0.28	-	0.4	-	0.34	-
Smarce1	gi|10181166	0.54(0.07)	0.54	0.44	0.43	0.49	0.49
Smarcd1	gi|1549249	0.29	0.54	0.67	0.36	0.48	0.45
Smarcb1	gi|6755578	0.35	0.25	0.47	0.35	0.41	0.30
Smarcd2	gi|57013098	0.07	0.14	0.21	0.07	0.14	0.11
**PRC1/mblr complex**							
Rnf2	gi|33563274	0.42	0.19	0.3	0.42	0.36	0.31
Phc1	gi|1490546	0.1	0.1	0.07	0.03	0.09	0.07
Mblr (Pcgf6)	gi|28076973	0.18	0.18	0.18	0.09	0.18	0.14
Rybp	gi|5381327	0.14	0.5	0.14	-	0.14	0.25
**Transcription factors**							
Sall4	gi|81913723	3.25(0.18)	2.35(0.18)	3.95(0.04)	1.22(0.18)	3.60	1.79
Ubp1	gi|134032032	6	8.77	7.32	4.31	6.66	6.54
Tcfcp2	gi|15628025	1.14	1.71	1.72	1.41	1.43	1.56
Lsd1	gi|51315882	0.37	0.53	0.58	0.11	0.48	0.32
Sall1	gi|14164331	0.33	0.3	0.24	0.15	0.29	0.23
Esrrb	gi|28277057	1.61	5.36	1.13	1.61	1.37	3.49
Smarca5	gi|14028669	0.28	0.25	0.25	0.03	0.27	0.14
Wdr5	gi|16554627	0.72	0.88	1.25	1.46	0.99	1.17
Peg10	gi|98985814	0.38(0.08)	0.38	0.26	0.19	0.32	0.29
Yeats2	gi|84794613	0.21	0.09	0.13	-	0.17	0.05
Hcfc1	gi|34328130	0.1	0.1	0.17	0.03	0.14	0.07
Zmym4	gi|167555112	0.08	0.1	0.19	0.08	0.14	0.09
Hells	gi|12232371	0.15	0.11	0.24	0.07	0.20	0.09
Mga	gi|120444914	0.06	0.04	0.1	0.01	0.08	0.03
Zfp462	gi|85740499	0.15	0.17	0.02	0.01	0.09	0.09
Oct4	gi|125490392	0.45	-	0.45	-	0.45	-
Requiem	gi|6648956	0.36	0.26	0.37	0.17	0.37	0.22
Pogz	gi|111598687	0.14	0.14	0.03	-	0.09	0.07
Zfp143	gi|121247390	0.28	0.16	0.28	0.17	0.28	0.17
Sin3a	gi|726286	0.1	0.12	0.1	-	0.10	0.06
Hnrnpab	gi|6754222	0.54	0.38	0.24	0.91	0.39	0.65
Wiz	gi|46909565	0.07	0.02	0.17	0.02	0.13	0.02
Adnp	gi|55930867	0.12	0.12	0.12	0.04	0.12	0.08
Satb2	gi|20982839	0.23	0.08	0.18	0.13	0.21	0.11
Klf5	gi|31981873	0.22	0.14	0.22	0.07	0.22	0.11
Trim33	gi|119637828	0.03	0.12	0.11	0.06	0.07	0.09
Mybl2	gi|6678974	0.14	0.14	0.09	0.09	0.12	0.12
Bptf	gi|123241372	0.02	0.01	0.03	-	0.03	0.01
Grhl2	gi|46810275	0.3	0.07	0.14	-	0.22	0.04
L3mbtl2	gi|27734414	0.13	0.04	0.04	-	0.09	0.02
Zzz3	gi|47847456	0.03	0.03	0.03	-	0.03	0.02
Ehmt1	gi|34784556	0.12	-	0.03	-	0.08	-
Zfp828	gi|32469497	0.05	-	0.08	-	0.07	-
**Other**							
Rif1	gi|47078460	0.32	0.34	0.48	0.29	0.40	0.32
Zcchc8	gi|169808385	0.6	0.75	0.83	-	0.72	0.38
Supt16h	gi|15637171	0.18	0.18	0.25	0.12	0.22	0.15
Lig3	gi|3913496	0.27	0.27	0.23	0.13	0.25	0.20
Ogt	gi|27499606	0.22	0.15	0.22	-	0.22	0.08
Xrcc6	gi|145587104	0.27	0.27	0.42	0.06	0.35	0.17
Xrcc5	gi|22137748	0.18	0.33	0.27	0.08	0.23	0.21
C130039O16Rik	gi|148670819	0.16	0.13	0.10	0.10	0.13	0.12
Msh2	gi|726086	0.17	0.17	0.25	-	0.21	0.09
L1td1	gi|124487095	0.21	0.3	0.12	0.08	0.17	0.19
Msh6	gi|6754744	0.12	0.02	0.07	-	0.10	0.01
Rbm14	gi|86262142	0.1	0.16	0.21	-	0.16	0.08
Xrcc1	gi|55391482	0.16	0.05	0.22	0.1	0.19	0.08
Polb	gi|21729749	0.55	0.42	0.3	0.25	0.43	0.34
EMSY	gi|124249084	0.08	0.11	0.08	0.11	0.08	0.11
Rpa1	gi|18390321	0.1	-	0.16	-	0.13	-
Prkdc	gi|124517706	0.01	-	0.01	-	0.01	-
4632411B12Rik	gi|37360322	0.07	0.04	0.04	-	0.06	0.02
Akap8	gi|5931618	0.09	0.09	0.05	-	0.07	0.05
Pnkp	gi|7108591	0.13	-	0.12	0.06	0.13	0.03
Asf1a	gi|13384964	0.15	0.15	0.15	0.15	0.15	0.15
Cabin1	gi|70995287	0.04	-	0.07	-	0.06	-
2310057J16Rik	gi|61213696	0.03	0.1	0.03	-	0.03	0.05
Ubqln4	gi|15805016	0.05	0.05	0.05	-	0.05	0.03

[a] equivalent to Table S6.

Table S9. Esrrb-interacting proteins: emPAI scores

		Flag#1	Flag#2	Flag#3	Flag#4		
		-Dox	+Dox	-Dox	+Dox	-Dox	+Dox
Protein	Accession	emPAI[a]	emPAI[a]	emPAI[a]	emPAI[a]	Average emPAI	
**Esrrb**	gi|6166153	29.49	55.48	29.03	26.16	29.26	40.82
**SWI/SNF complex**							
Smarcc1	gi|30851572	0.86(0.03)	0.76(0.06)	1.01	0.48	0.94	0.62
Arid1a	gi|124249109	0.06(0.01)	0.09	0.22	0.05	0.14	0.07
Smarca4	gi|76253779	0.35	0.23	0.34	0.2	0.35	0.22
Smarcd1	gi|1549249	0.85	0.47	0.77	0.69	0.81	0.58
Pbrm1	gi|116284015	0.07	0.03	0.13	0.07	0.10	0.05
**Trrap/p400 complex**							
Trrap	gi|124486949	0.19	0.16	0.45	-	0.32	0.08
Ep400	gi|27348237	0.14	0.13	0.25	0.02	0.20	0.08
Yeats4	gi|13386064	1.41	1.41	0.47	-	0.94	0.71
Dmap1	gi|12963557	0.46	0.29	0.39	0.07	0.43	0.18
Brd8	gi|19344050	0.2	-	0.15	0.04	0.18	0.02
Ing3	gi|17390799	0.16	0.16	0.25	-	0.21	0.08
1600027Rik	gi|110625965	0.15	0.15	0.16	-	0.16	0.08
**NuRD complex**							
Chd4	gi|39204553	0.39	0.22	0.25	0.1	0.32	0.16
Gatad2a	gi|148696823	0.42	1.12	0.67	0.71	0.55	0.92
Mta2	gi|51491880	0.43	0.5	0.52	0.27	0.48	0.39
Mta1	gi|86577662	0.29	0.18	0.3	0.15	0.30	0.17
Gatad2b	gi|21314854	0.36	0.59	0.45	0.18	0.41	0.39
Mta3	gi|18381007	0.1	0.28	0.17	-	0.14	0.14
Mbd3	gi|7305261	0.68	0.86	0.7	0.12	0.69	0.49
Hdac1	gi|2347180	1.64(0.2)	2.17(0.13)	1.26(0.06)	1.04	1.45	1.61
Hdac2	gi|87162464	0.62	0.72	1.4(0.06)	0.68	1.01	0.70
Rbbp4	gi|5032027	0.79(0.14)	0.68(0.21)	0.49(0.14)	0.41(0.3)	0.64	0.55
**Mediator complex**							
Med14	gi|115270972	0.77	1.17	1.06	0.75	0.92	0.96
Med12	gi|123226656	0.32	0.4	0.48	0.31	0.40	0.36
Med23	gi|61651678	0.38	0.75	0.81	0.47	0.60	0.61
Med24	gi|119220579	0.73	0.73	0.74	0.45	0.74	0.59
Med17	gi|21450345	1.41	2.34	1.05	1.2	1.23	1.77
Med1	gi|14193713	0.25	0.42	0.56	0.36	0.41	0.39
Med16	gi|148699683	0.63	0.69	0.54	0.51	0.59	0.60
Med15	gi|32451779	0.86	0.79	0.75	0.58	0.81	0.69
Med13	gi|124286862	0.13	0.12	0.18	0.06	0.16	0.09
Med27	gi|16741439	3.94	2.74	1.16	0.98	2.55	1.86
Med25	gi|47940179	0.41	0.41	0.31	0.38	0.36	0.40
Med13l	gi|49257394	0.1	0.12	0.14	0.08	0.12	0.10
Med4	gi|13385626	0.75	4.36	2.17	1.6	1.46	2.98
Med26	gi|28466971	0.37	0.6	0.46	0.09	0.42	0.35
Med6	gi|27754027	1.79	1.41	1.13	2.48	1.46	1.95
Med8	gi|29366816	0.94	1.42	0.77	0.6	0.86	1.01
Cncc	gi|38382739	0.43	0.88	0.45	0.1	0.44	0.49
Med30	gi|19882231	1.24	1.24	0.39	0.19	0.82	0.72
Med7	gi|157266302	0.84	0.84	0.46	0.14	0.65	0.49
Med29	gi|27754101	1.17	0.86	0.38	0.39	0.78	0.63
Cdk8	gi|31652272	0.13	0.07	0.14	0.14	0.14	0.11
Med18	gi|21313064	0.75	1.01	0.16	0.81	0.46	0.91
Med19	gi|28277157	0.29	1.43	0.14	0.14	0.22	0.79
**RNApol2 complex**							
Polr2a	gi|2145091	0.22	0.21	0.27	0.2	0.25	0.21
Polr2b	gi|24418911	0.26	0.22	0.52	0.31	0.39	0.27
Polr2c	gi|29336059	0.69	0.69	0.39	0.25	0.54	0.47
Polr2g	gi|4505947	0.19	1.34	0.69	0.2	0.44	0.77
**TFIID complex**							
Taf9	gi|28175808	0.41	1.23	1.58	0.13	1.00	0.68
Taf6	gi|6678215	0.1	0.1	0.1	0.05	0.10	0.08
Taf4a	gi|123288532	0.1	0.05	0.28	0.11	0.19	0.08
Taf10	gi|46518499	0.16	0.16	0.17	-	0.17	0.08
Tbp	gi|29477183	0.1	0.1	0.1	-	0.10	0.05
**TRX/MLL complex**							
Wdr5	gi|16554627	0.72	0.72	0.59	0.47	0.66	0.60
Hcfc1	gi|34328130	0.08	-	0.12	0.05	0.10	0.03
Mll2	gi|149266757	0.01	0.01	0.05	-	0.03	0.01
Mll3	gi|37999865	0.01	0.01	0.04	-	0.03	0.01
Ashl2	gi|4009338	0.1	0.05	0.29	-	0.20	0.03
Rbbp5	gi|34784634	0.06	0.06	0.19	-	0.13	0.03
**Transcription factors**							
Sall4	gi|117553631	0.92(0.13)	0.75(0.13)	0.58(0.18)	0.56(0.09)	0.75	0.66
Dax1	gi|6671531	1.41	0.87	1.04	0.5	1.23	0.69
Tcfcp2l1	gi|90101766	0.96	0.63	1	0.48	0.98	0.56
Fkbp15	gi|38614309	0.25	0.32	0.3	0.14	0.28	0.23
Esrra	gi|112293262	0.67	1.59	1.29	0.48	0.98	1.04
Ncoa3	gi|118026946	0.12	0.05	0.23	-	0.18	0.03
Ubp1	gi|134032032	0.48	0.71	0.5	0.07	0.49	0.39
Nrip1	gi|27734110	0.17	0.45	0.25	0.37	0.21	0.41
Sall1	gi|14164331	0.13	0.1	0.25	0.11	0.19	0.11
Zfp462	gi|148670321	0.02	0.02	0.14	-	0.08	0.01
Cdc2a	gi|13542826	0.81	0.81	1.03	0.11	0.92	0.46
Zbtb9	gi|54400753	0.4	0.31	0.62	0.15	0.51	0.23
Smarca5	gi|14028669	0.09	0.06	0.19	0.23	0.14	0.15
Wiz	gi|46909565	0.03	0.02	0.18	0.11	0.11	0.07
Requiem	gi|6648956	0.17	0.26	0.36	0.09	0.27	0.18
Jmjd1c	gi|149260924	0.03	0.01	0.04	-	0.04	0.01
Tcfcp2	gi|15628025	0.12	0.19	0.2	-	0.16	0.10
L3mbtl2	gi|27734414	0.07	-	0.16	0.05	0.12	0.03
Oct4	gi|125490392	0.1	-	0.33	-	0.22	-
Myst1	gi|21312790	0.07	0.07	0.14	-	0.11	0.04
Ehmt1	gi|34784556	0.03	0.03	0.1	-	0.07	0.02
**Other**							
Rif1	gi|47078460	0.39	0.26	0.41	0.06	0.40	0.16
Ogt	gi|27499606	0.3	0.15	0.39	0.1	0.35	0.13

[a] equivalent to Table S6.

Table S9, related to Figure 2
